# Light amplified oxidative stress in tumor microenvironment by carbonized hemin nanoparticles for boosting photodynamic anticancer therapy

**DOI:** 10.1038/s41377-021-00704-5

**Published:** 2022-03-01

**Authors:** Liyun Lin, Wen Pang, Xinyan Jiang, Shihui Ding, Xunbin Wei, Bobo Gu

**Affiliations:** 1 Med-X Research Institute and School of Biomedical Engineering, Shanghai Jiao Tong University, Shanghai, 200030 China; 2 Biomedical Engineering Department, Peking University, Beijing, 100081 China; 3 Key Laboratory of Carcinogenesis and Translational Research (Ministry of Education/Beijing), Peking University Cancer Hospital & Institute, Beijing, 100142 China

**Keywords:** Biophotonics, Biomedical materials

## Abstract

Photodynamic therapy (PDT), which utilizes light excite photosensitizers (PSs) to generate reactive oxygen species (ROS) and consequently ablate cancer cells or diseased tissue, has attracted a great deal of attention in the last decades due to its unique advantages. However, the advancement of PDT is restricted by the inherent characteristics of PS and tumor microenvironment (TME). It is urgent to explore high-performance PSs with TME regulation capability and subsequently improve the therapeutic outcomes. Herein, we reported a newly engineered PS of polymer encapsulated carbonized hemin nanoparticles (P-CHNPs) via a facile synthesis procedure for boosting photodynamic anticancer therapy. Solvothermal treatment of hemin enabled the synthesized P-CHNPs to enhance oxidative stress in TME, which could be further amplified under light irradiation. Excellent in vitro and in vivo PDT effects were achieved due to the improved ROS (hydroxyl radicals and singlet oxygen) generation efficiency, hypoxia relief, and glutathione depletion. Moreover, the superior in vitro and in vivo biocompatibility and boosted PDT effect make the P-CHNPs a potential therapeutic agent for future translational research.

## Abstract (web summary)

We reported a newly engineered photosensitizer of polymer encapsulated carbonized hemin nanoparticles (P-CHNPs) via a facile synthesis procedure for boosting photodynamic therapy (PDT). The synthesized P-CHNPs achieved enhanced oxidative stress in tumor microenvironment, which could be further amplified under light irradiation, enabling excellent in vitro and in vivo PDT effects. Moreover, the superior in vitro and in vivo biocompatibility and boosted PDT effect make the P-CHNPs a potential therapeutic agent for future translational research.

## Graphical abstract

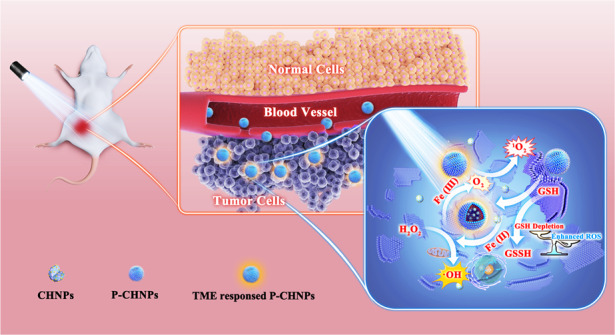

## Introduction

Cancer, the second leading cause of death currently, is one major public health issue with the increased incidence rate in the past several decades^1^. Various emerging cancer treatment strategies, including gene therapy^2^, photodynamic therapy (PDT)^3–5^, photothermal therapy (PTT)^6–8^, chemodynamic therapy (CDT), etc., have been proposed and demonstrated. Among these newly developed treatment strategies mentioned above, PDT, which utilizes photosensitizer (PS) in the presence of light with a specific wavelength to generate reactive oxygen species (ROS) to irreversibly destroy the targeted diseased tissue, is recognized as a promising treatment strategy with various benefits including noninvasiveness, low side-effect, high spatial resolution, etc.^9–11^. Since approved for clinical use for the first time to treat bladder cancer in 1993, PDT has been clinically applied to treat various types of cancers^12^. However, PDT has not yet reached its full potential due to the limitation from PSs and tumor microenvironment (TME). Recently, many new PSs with improved ROS generation capability including fluorogens with aggregation-induced emission (AIE) characteristics (AIEgens)^13,14^, graphene quantum dots (GQDs)^15^, black phosphorus^16^, carbon dots (CDs)^17^, etc., have been designed and synthesized for PDT applications. The ROS quantum yield of GQDs could even reach 1.3^18^. Although the inherent ROS generation efficiency of emerging PSs was significantly enhanced, the ROS quantity in tumor site and consequently PDT efficiency were still subject to the TME features^19^, e.g., limited ROS quantity due to the hypoxia in the tumor site, scavenged ROS in cancer cells due to the overexpressed glutathione (GSH) of a tumor, etc. Thus, synergistic therapies such as PDT/PTT^20^, PDT/chemotherapy^21^, PDT/immunotherapy^16^, and PDT/CDT^22^, were employed to improve the therapeutic outcomes. It is worth to note that TME is featured with H_2_O_2_ overexpression (0.1 to 1 mM), low catalase activity, mild acidity^23^. In the CDT process, under the assistant of Fenton ions (Fe^2+^) or other Fenton-like ions, H_2_O_2_ could be catalyzed via Fenton/Fenton-like reaction to generate cytotoxic hydroxyl radicals (•OH), one kind of ROS, and subsequently damage the tumors^24^. Recent advances showed that Fenton/Fenton-like reaction could produce both cytotoxic •OH and oxygen (O_2_), and the quantity of both •OH and O_2_ could be significantly enhanced under light irradiation via photo-Fenton reactions^25^. It means that endogenous H_2_O_2_ can be decomposed to more •OH and O_2_ once light irradiation was employed to accelerate Fenton-like reaction. Therefore, CDT could relieve O_2_ deficiency in PDT and improve therapeutic effect, moreover, light irradiation could improve •OH and O_2_ generation efficiency to enhance antitumor effects of CDT.

Considering that Fenton/Fenton-like reactions could improve the anticancer performance of PDT by amplifying the oxidative stress in TME, various therapeutic nanoagents have been designed and synthesized for CDT/PDT synergistic therapy. These efforts were mainly focused on functionalizing PSs with H_2_O_2_-responsed catalysts/enzymes such as Mn, Cu, Fe, Co, Pt-contained compounds to realize “all-in-one” multifunction^24^. The synthesized MSNs@CaO_2_-ICG@LA nanoagent^22^, UCNPs@TiO_2_@MnO_2_ core/shell/sheet nanocomposites^26^, and Au_2_Pt-PEG-Ce6 nanoformulation^27^, etc., achieved good anticancer performance, but these therapeutic nanoagents suffered from complicated synthesis and postprocessing procedures, and termination of ROS generation in TME. In order to further enhance the efficiency of CDT/PDT, some therapeutic agents with light amplified oxidative stress capability, such as UCNPs@MnSiO_3_@g-C_3_N_4_ nanoplatforms^28^, Cu(II)-g-C_3_N_4_ nanosheets^29^, manganese ferrite nanoparticle, and chlorin e6 (Ce6) anchored mesoporous silica nanoparticles (MFMSNs)^30^, and copper ferrite nanospheres^23^, have been designed and synthesized. However, these therapeutic nanoagents suffer from not only complicated synthesis and/or postprocessing procedures but also limited synergistic CDT/PDT effects. Moreover, various heavy metal ions were contained in the synthesized nanoagents, potentially increasing unpredictable toxicity and subsequently limitation for their future clinical applications. It is urgent to develop superior biocompatible PSs via simple synthesis to efficiently amplify oxidative stress in TME and subsequently boost the anticancer performance of PDT.

Carbon nanomaterials, featured with superior biocompatibility, have been used as phototherapy (including PDT and PTT) and optical imaging nanoagents^31^. Most carbon nanoagents were commonly synthesized by simple carbonization of inert raw materials, indicating that carbonization could endow them with new functions. Thus, the carbonization of appropriate functional materials paves a new avenue to synthesize PSs with TME regulation capability. Hemin, which is Fe(III)-contained protoporphyrin IX and usually acts as the catalytic center of many protein families including cytochromes and hemoglobins, can catalyze various oxidation reactions to decompose H_2_O_2_ to O_2_/ **∙**OH^32^. In addition, hemin tends to lose the iron in the porphyrin ring under acidic or high-temperature conditions to become porphyrin, which has high singlet oxygen (^1^O_2_) generation efficiency^33^. All these characteristics make hemin a good candidate for carbonization to form PS with oxidative stress amplification capability in TME and superior biocompatibility. However, most efforts have been devoted to packaging, carrying, and decorating with other materials to improve the dispersion of hemin for efficient catalytic activities up to now^34–36^. There were no reports about rational and facile synthesis methods to form PS, which could boost the PDT effect, using hemin as raw materials.

**Scheme 1:**
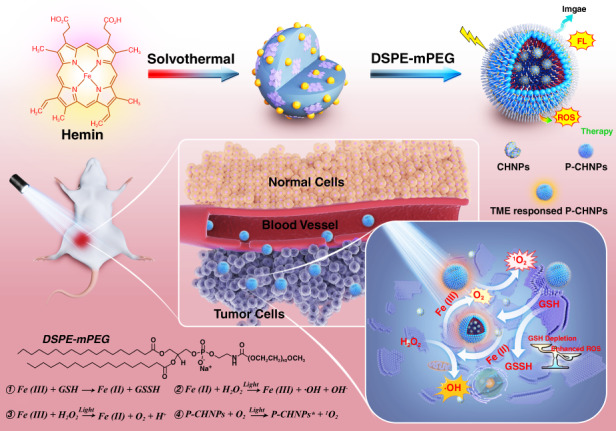
Scheme of the synthesis approach of P-CHNPs and therapeutic mechanism of light amplified oxidative stress in TME by P-CHNPs for boosting photodynamic anticancer therapy

Here, we reported a rationally engineered PS with TME regulation capability and superior biocompatibility. Carbonized hemin nanoparticles (CHNPs) were designed and synthesized via facile solvothermal carbonization reaction and then encapsulated with an amphiphilic molecule to obtain polymer encapsulated carbonized hemin nanoparticles (P-CHNPs). As shown in Scheme 1, the proposed P-CHNPs could efficiently amplify the oxidative stress in TME. The endogenous H_2_O_2_ was catalyzed to O_2_ and •OH via Fenton/Fenton-like reactions to efficiently relieve hypoxia and improve ROS generation within the tumor site. Meanwhile, GSH depletion impaired the antioxidant defense system and remarkably enhanced the anticancer effect. Moreover, the oxidative stress in TME could be amplified with the aid of light irradiation, boosting PDT efficiency. It is worth to note that the superior in vitro and in vivo biocompatibility and boosted PDT effect of P-CHNPs guarantee the feasibility in anticancer therapy and future translational research.

## Results

### Synthesis and characterization of P-CHNPs

**Fig. 1:**
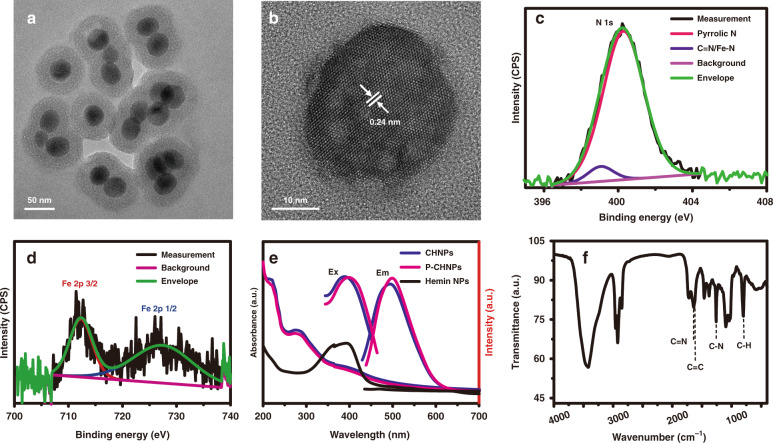
The characterization of P-CHNPs. **a** The typical TEM image of the synthesized P-CHNPs. **b** The HRTEM image of the obtained P-CHNPs and the corresponding crystal lattice spacing values. The high-resolution XPS of P-CHNPs for **c** N 1*s* and **d** Fe 2*p*. **e** The UV-vis absorption, excitation (Ex), and emission (Em) spectra of hemin nanoparticles (NPs), CHNPs, and P-CHNPs. [CHNPs] = 50 μg mL^−1^, [P-CHNPs] = 50 μg mL^−1^. **f** FT-IR spectrum of P-CHNPs

The CHNPs were prepared by the solvothermal carbonized reaction using hemin as the raw materials. The size and morphology of the synthesized nanoparticles were characterized by transmission electron microscopy (TEM) and dynamic light scattering (DLS) measurement. The TEM images showed that the CHNPs had dispersive and uniform morphology (Figs. S1, S2a). The DLS results showed that the average hydrodynamic diameter of CHNPs was about 44 nm (Fig. S2b). In order to further improve the biocompatibility, CHNPs were encapsulated with amphiphilic polymer DSPE-mPEG to form P-CHNPs. The TEM images indicated that the P-CHNPs exhibited a core-shell structure with an average diameter of 73 nm (Fig. 1a and Fig. S2c), and the DLS result was about 79 nm (Fig. S2d). The structural details of P-CHNPs were further investigated with high-resolution TEM (HRTEM) as shown in Fig. 1b. The high crystallinity with a lattice spacing of 0.24 nm ascribed to the (100) lattice plane of graphene was observed^37^. Then high-angle annular dark-field scanning TEM (HAADF-STEM) was applied to study the elemental composition and distribution of P-CHNPs. As shown in Fig. S3, the characteristic element P of DSPE-mPEG and element O were homogeneously distributed in the core region and in the whole nanoparticle respectively, demonstrating that CHNPs were encapsulated by DSPE-mPEG to form core-shell structure through hydrophobic interaction and consequently protect and stabilize the CHNPs. Fe, the characteristic element of hemin, was homogeneously distributed in the whole nanoparticle, indicating that Fe was released from hemin during solvothermal deferrization reaction and subsequently combined with O, N-containing functional groups existed in the whole nanoparticle. The homogeneously distributed Fe, which is favorable for electronic exchange with substrates such as H_2_O_2_, facilitates the Fenton/Fenton-like reaction. Furthermore, the energy-dispersive X-ray spectroscopy (EDX) revealed the existence and content of C, N, O, P, and Fe elements as shown in Fig. S4. The content of Fe in P-CHNPs reached a high level of 13.36 wt%, endowing it with considerable active sites density. Then the chemical composition and elementary chemical form of P-CHNPs were studied by X-ray photoelectron spectroscopy (XPS) and high-resolution XPS. Five typical peaks of C 1*s* (284.1 eV), N 1*s* (398.5 eV), O 1*s* (531.1 eV), P 2*p* (144.2 eV), and Fe 2*p* (727.4 eV)^38^ were presented as shown in Fig. S5a. The C 1*s* displayed four characteristic peaks: C–C (284.2 eV), C–N (284.8 eV), C–O (286.2 eV), and C=O (288.8 eV)^38^ (Fig. S5b), indicating that there were O, N-containing groups to combine the released Fe ion stemming from hemin deferrization. While the characteristic peaks of N 1*s* located at 399.1 and 400.2 eV (Fig. 1c), correspond to C=N/Fe–N and pyrrolic N respectively^39^. It means that there were coordination interactions between Fe and N-contained groups, enabling it to perform Fenton/Fenton-like reaction and generate ^1^O_2_ simultaneously^40^. Furthermore, the chemical form of the iron element, the key element for Fenton/Fenton-like reaction, was also analyzed as shown in Fig. 1d. The characteristic peaks of Fe 2*p*, i.e., Fe 2*p*_3/2_ (712.4 eV) and Fe 2*p*_1/2_ (728.8 eV), indicated the presence of Fe (III)^41^.

In order to study the new functions endowed by carbonization, the hemin nanoparticles (Hemin NPs, hemin was encapsulated with DSPE-mPEG) were also prepared for comparison. The UV-Vis absorption spectra of the synthesized Hemin NPs, CHNPs, and P-CHNPs were measured as shown in Fig. 1e. The Hemin NPs showed obvious characteristic absorption peaks at about 400 nm attributable to the raw materials hemin. It was observed that both CHNPs and P-CHNPs had main absorption peaks at 210 and 270 nm, which were derived from the π-π* transition of aromatic C=C bonds and n-π* transition of the functional groups with lone pairs electron, respectively^42^. Meanwhile, it also showed a broad absorption extended to 700 nm, which might originate from the functionalized surface groups of the nanoparticles^43^, and the shoulder peak at 404 nm likely originated from the noncompletely carbonized hemin/porphyrin ring^44^. The P-CHNPs had excitation-dependent emission features (Fig. S6) and the measured fluorescence quantum yield was 0.016 using riboflavin (Φ_riboflavin_ = 0.23 in DMSO^45^) as the reference, enabling image-guided anticancer therapy, while the raw material hemin had no fluorescence emission. Moreover, the encapsulation with DSPE-mPEG had no significant influence on the spectra of the synthesized CHNPs. Then the functional groups of P-CHNPs were analyzed using the Fourier transform infrared (FT-IR) spectroscopy as shown in Fig. 1f. In addition to the vibration bands of O–H/N–H, -CH_3_/-CH_2_, C–N, some characteristic vibrations were also recorded. Two FT-IR peaks at 1639 and 1609 cm^−1^ could be ascribed to the C=N and C=C stretching vibrations of the porphyrin ring, while the peak at 803 cm^−1^ indicated the presence of C–H out-of-plane ring deformation corresponding to pyrrole ring^46,47^. It can be deduced that not all the porphyrin rings were carbonized by the solvothermal reaction, enabling P-CHNPs to generate ROS as well as regulate TME. Moreover, the O, N-containing functional groups further improved the hydrophilicity and stability of the synthesized P-CHNPs in aqueous solutions.

### TME regulation by P-CHNPs

**Fig. 2:**
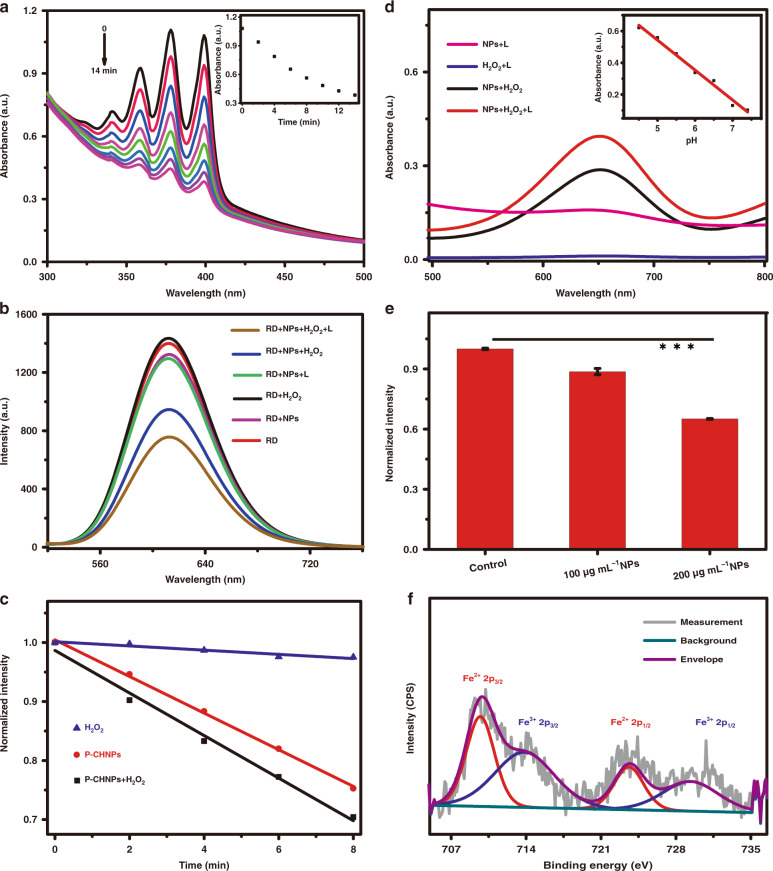
The oxidative stress-related properties of P-CHNPs. **a** The absorption spectra of the mixture solution of ABDA and P-CHNPs under light irradiation for different time. Inset: absorption variation of mixture solution at 399 nm with the increment of irradiation time. [P-CHNPs] = 50 μg mL^−1^, [ABDA] = 50 μM. **b** The fluorescence spectra of RDPP in different mixture solutions. [P-CHNPs] = 100 μg mL^−1^, [RDPP] = 30 μM. **c** The consumption rate of ABDA in different mixture solutions under light irradiation. [ABDA] = 50 μM, [P-CHNPs] = 50 μg mL^−1^. **d** UV-vis absorption spectra of TMB in different mixture solution (pH 6.5) under light irradiation. Inset: absorption variation (650 nm) of TMB, H_2_O_2_ and P-CHNPs mixture solution with different pH values. [P-CHNPs] = 50 μg mL^−1^, [TMB] = 800 μM, [H_2_O_2_] = 300 μM. **e** Optical density value variations of GSH Colorimetric Assay Kit treated with different concentrations of P-CHNPs. ***P < 0.001, *n* = 5. **f** The high-resolution XPS of P-CHNPs reacted with GSH for Fe 2*p*. L: light irradiation (400–700 nm, 70 mW cm^−2^, 20 min), NPs: P-CHNPs, RD: RDPP

Since the facile solvothermal reaction endowed P-CHNPs with new functions, the ROS generation and TME regulation capability of P-CHNPs as well as the amplification by light irradiation were characterized as shown in Fig. 2. ^1^O_2_ generation efficiency of P-CHNPs in an aqueous medium (50 μg mL^−1^) was evaluated under the light irradiation (400–700 nm, 70 mW cm^−2^) using 9,10-anthracenediyl-bis(methylene)dimalonic acid (ABDA) (50 μM) as the ^1^O_2_ indicator. As shown in Fig. 2a, the ABDA was almost consumed up in the presence of P-CHNPs after 14 min illumination, while in the absence of P-CHNPs, the absorption of ABDA was kept constant under the same light irradiation (Fig. S7), eliminating interference from light irradiation. Moreover, the ^1^O_2_ generation capability of hemin, the raw materials of P-CHNPs, was also investigated, and no obvious consumption of ABDA was observed, indicating that not raw materials but solvothermal treatment endows P-CHNPs with the ^1^O_2_ generation capability. In order to quantify ^1^O_2_ generation efficiency, one commercial photosensitizer Ce6 (Φ_Ce6_ = 0.66 in aqueous media^48^) was selected as the reference. As shown in Fig. S8, the ^1^O_2_ generation capability of the synthesized P-CHNPs was measured as 0.35, which was much higher than that of one US Food and Drug Administration (FDA) approved PS, indocyanine green (ICG, Φ_ICG_ = 0.002)^49^.

Since H_2_O_2_ is overexpressed in TME, which could be regulated by P-CHNPs via Fenton/Fenton-like activity, the catalytic reaction products of H_2_O_2_, i.e., O_2_ and •OH, were measured to study the oxidative stress in TME regulated by P-CHNPs. [Ru(dpp)_3_]Cl_2_ (RDPP), which fluorescence can be immediately quenched by O_2_^50^, was selected as the indicator to monitor the generation of O_2_. As shown in Fig. 2b, the fluorescence intensity of RDPP was significantly decreased when RDPP was mixed with both P-CHNPs and H_2_O_2_, while no remarkable fluorescence intensity variation was observed when RDPP was mixed with either P-CHNPs or H_2_O_2_ alone. It can be inferred that H_2_O_2_ could be catalyzed by P-CHNPs to generate O_2_ to quench the fluorescence of RDPP. Moreover, the fluorescence intensity of RDPP was further decreased when light irradiation (400–700 nm, 70 mW cm^−2^, 20 min) was employed, indicating that light irradiation could efficiently enhance the generation of O_2_. Then, the influence of enhanced O_2_ generation on ^1^O_2_ production was studied by the ABDA degradation experiments. As shown in Fig. 2c, the degradation of ABDA was significantly expedited when H_2_O_2_ was added to the mixture solution of ABDA and P-CHNPs, while the H_2_O_2_ alone had no obvious influence on the degradation of ABDA. It means that more O_2_ supplied by the Fenton-like reaction remarkably improved the generation efficiency of ^1^O_2_, and subsequently amplified the oxidative stress regulation capacity in TME. Meanwhile, the •OH generation capability, product of TME regulation via Fenton reaction under the assistant of P-CHNPs, was studied in H_2_O_2_ solution using 3,3′,5,5′-tetramethylbenzidine (TMB) as the chromogenic substrate, which could be oxidized by the highly reactive •OH to appear an absorption peak at 650 nm. As shown in Fig. S9a, when TMB was mixed with either P-CHNPs or H_2_O_2_ alone under mild acidic conditions (pH = 6.5), which was similar to the pH value in TME, no obvious absorption at 650 nm was detected even the reaction time of the mixture solution reached 1 h. While the strong absorption peak at 650 nm was recorded once the TMB was mixed with both P-CHNPs and H_2_O_2_, indicating efficient •OH generation via catalyzing H_2_O_2_ by P-CHNPs. Considering that TME was featured with H_2_O_2_ overexpression and mild acidity (typically around 6.5), the catalytic activity of P-CHNPs was investigated under different pH conditions to evaluate its oxidative stress regulation capability in TME. As shown in the insets of Figs. 2d and S9b, the characteristics absorption of TMB at 650 nm grew stronger with the decrement of pH value from 7.4 to 4.5, which was accompanied by the color changes from colorless to blue. It indicated that P-CHNPs possessed regulation capability in mild acidic (pH = 6.5) condition which is similar to TME acidity and were inactive in neutral (pH = 7.4) condition, enabling P-CHNPs to selectively regulate oxidative stress in TME. Moreover, the characteristic absorption of TMB grew stronger when light irradiation (400–700 nm, 70 mW cm^−2^, 20 min) was employed (Fig. 2d), indicating that light could efficiently enhance the H_2_O_2_ decomposition to more lethal •OH.

In order to further confirm the efficient •OH and ^1^O_2_ generation capability of P-CHNPs, electron spin resonance (ESR) analysis was employed using 5,5-dimethyl-1-pyrroline-*N*-oxide (DMPO) and 2,2,6,6-tetramethylpiperidine (TEMP) as •OH-trapping agent and ^1^O_2_-trapping agent, respectively. The typical 1:2:2:1 ESR signal of •OH (Fig. S10a) confirmed the generation of •OH via catalyzing H_2_O_2_ by P-CHNPs. And the ESR signal was significantly enhanced once light irradiation was applied, indicating that light promoted the H_2_O_2_ decomposition to •OH via photo-Fenton reaction. When DMPO was mixed with either P-CHNPs or H_2_O_2_ alone with/out light irradiation, no characteristic signal was detected. The measured 1:1:1 ESR signal of ^1^O_2_ (Fig. S10b) validated that P-CHNPs could generate ^1^O_2_ under light irradiation, while no ESR signal was detected from P-CHNPs without light irradiation. To further verify that P-CHNPs could catalyze H_2_O_2_ to O_2_ and subsequently amplify ^1^O_2_, H_2_O_2_ was added to the sample, then enhanced ESR signal was detected, while no ESR signal was detected from H_2_O_2_ under light irradiation. These results indicated that light irradiation could enhance the generation capability of •OH and ^1^O_2_, which matched well with the experimental results in Fig. 2c, d.

As the well-known intracellular antioxidant, GSH, which can protect cells, is overexpressed in cancer cells and increases resistance to PDT. Thus, reduction of intracellular GSH level is of great importance for improving the anticancer performance of PDT. Considering that the Fe (III) in P-CHNPs could react with GSH, significantly lowering the GSH level, the GSH Colorimetric Assay Kit, which optical density value varies with the GSH content, was selected to investigate the GSH depletion capability of P-CHNPs. As shown in Fig. 2e, the optical density value decreased with the increment of P-CHNPs concentration, indicating that GSH was consumed by P-CHNPs. Meanwhile, the reaction products were also monitored with XPS as shown in Fig. 2f. Once reacted with GSH, the Fe 2p spectrum of P-CHNPs displayed two new characteristic peaks at 709 and 724 eV belonging to Fe (II) (red lines), which indicated that part of Fe (III) in P-CHNPs was reduced to catalytic Fe (II). These results showed that the reaction products of GSH and P-CHNPs contained Fe of mixed-valence states. Thus, GSH was efficiently depleted by P-CHNPs, enhancing the oxidative stress and therapeutical outcomes.

### The mechanism of light amplified oxidative stress in TME

The mechanism of light amplified oxidative stress in TME by P-CHNPs was summarized in Scheme 1. The Fe (III) in P-CHNPs could catalyze H_2_O_2_ decomposition and be reduced to catalytic Fe (II) to form the Fe (III)/Fe (II) redox switch, but the Fenton-like reaction efficiency was very limited. Once light irradiation was employed, photoreduction of Fe (III) to Fe (II) was expedited, enabling the cycle of Fe (III) and Fe (II). It means that the Fe (III)/Fe (II) redox switch is triggered by light irradiation and the termination of TME regulation via Fenton/Fenton-like reactions is efficiently avoided^51^. Meanwhile, the continuous Fenton/Fenton-like reactions catalyzed H_2_O_2_ decomposition to generate cytotoxic •OH and O_2_ with remarkably improved generation efficiency. Additionally, the P-CHNPs acted as efficient PS for PDT, hypoxia-induced limitation on the PDT effect was eliminated due to the self-supplied O_2_ from TME regulation. GSH, overexpressed in tumor cells could reduce Fe (III) to catalytic Fe (II). Once GSH was depleted, the oxidative stress could be amplified and consequently enhance the anticancer effect. Overall, light could further amplify TME-mediated redox reaction and enable high generation efficiency of •OH and ^1^O_2_, hypoxia relief, and GSH depletion, leading to boosted photodynamic anticancer therapy via amplified oxidative stress in TME.

### In vitro biocompatibility and intracellular ROS generation capability of P-CHNPs

Before performing the in vitro study, the stability of P-CHNPs was evaluated by measuring the UV-vis absorption spectra. The P-CHNPs showed excellent physiological stability as the absorption peaks at 404 nm exhibited negligible fluctuations when incubated in different physiological solutions including phosphate-buffered saline (PBS) and cell medium (1640, DMEM) with 10% serum for up to 48 h (Fig. S11). The in vitro biocompatibility of P-CHNPs was first investigated by hemolysis assay. As shown in Fig. S12, no significant hemolysis (<5%) was observed when P-CHNPs were incubated with red blood cells (RBCs) for 12 h.

**Fig. 3:**
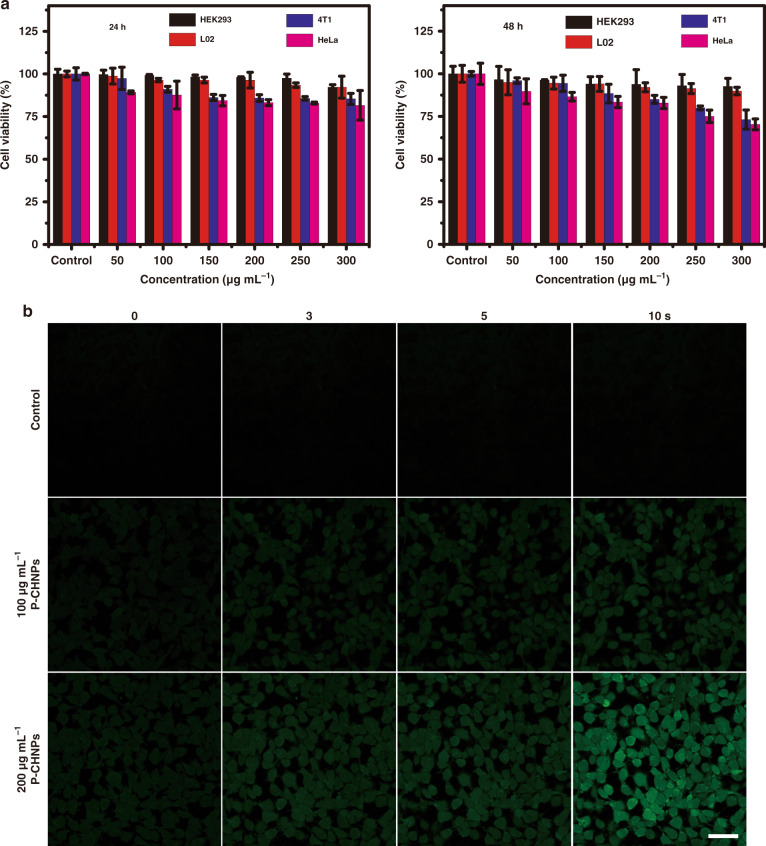
Cell viability and intracellular ROS generation capability of P-CHNPs. **a** Cell viability of 4T1 and HeLa cancer cells, HEK293, and L02 normal cells treated with various concentrations of P-CHNPs for 24 and 48 h. Data were expressed as means ± s.d. (*n* = 3). **b** Study of the intracellular ROS generation capability of various concentrations of P-CHNPs under light irradiation (400–700 nm, 200 mW cm^−2^). [DCFH-DA] = 5 μM, DCF (Ex: 488 nm, Em: 500–550 nm). Scale bar: 50 μm

In order to further study the biocompatibility and specific oxidative stress regulation in cancer cells, various concentrations of P-CHNPs were incubated with normal cells and cancer cells for different incubation times respectively, and the cell viability was evaluated using standard Cell Counting Kit-8 (CCK-8). As shown in Fig. 3a, there was no significant difference between the control cells and normal cells treated with P-CHNPs in the concentration range of 50–300 μg mL^−1^ even for 48 h, demonstrating the excellent biocompatibility. While the cell viability was remarkably decreased for cancer cells with an increment of concentration of P-CHNPs from 0 to 300 μg mL^−1^, indicating efficient •OH generation in cancer cells. It means that P-CHNPs could decompose the endogenous H_2_O_2_ under mild acidity conditions while being inactive to a neutral condition in normal cells. The hypoxia relief capability of P-CHNPs was studied in 4T1 cells by selecting RDPP as the O_2_ level indicator (Fig. S13). As compared with the 4T1 cells treated with RDPP alone or with RDPP/light irradiation, the 4T1 cells treated with RDPP and P-CHNPs showed weaker fluorescent intensity. And the fluorescent intensity was further decreased when 300 μM H_2_O_2_ was incubated with 4T1 cells treated with RDPP and P-CHNPs, indicating intracellular O_2_ supply by P-CHNPs via Fenton-like reaction. Moreover, when light irradiation was applied in the 4T1 cells treated with RDPP/P-CHNPs or RDPP/P-CHNPs/H_2_O_2_, the fluorescence intensity was decreased or completely quenched, these results clearly demonstrated that light irradiation promoted in situ H_2_O_2_ decomposition and intracellular O_2_ generation via P-CHNPs, efficiently overcoming tumor hypoxia.

The intracellular ROS generation capability of P-CHNPs was studied using 2′,7′-dichlorofluorescein diacetate (DCFH-DA) as the indicator. After incubating either with both of P-CHNPs and DCFH-DA or with DCFH-DA alone, the treated 4T1 cells were irradiated and imaged with confocal laser scanning microscopy (CLSM). As shown in Fig. 3b, after 10 s and 5 s light irradiation, a strong fluorescent signal of dichlorofluorescein (DCF) was observed within 4T1 cells treated with P-CHNPs with the concentration of 100 and 200 μg mL^−1^ respectively, demonstrating efficient and fast ROS generation capability of P-CHNPs. In contrast, no DCF signal was observed in the control cells, which eliminated any interference from light irradiation.

### The fluorescence imaging and in vitro anticancer effect of P-CHNPs

**Fig. 4:**
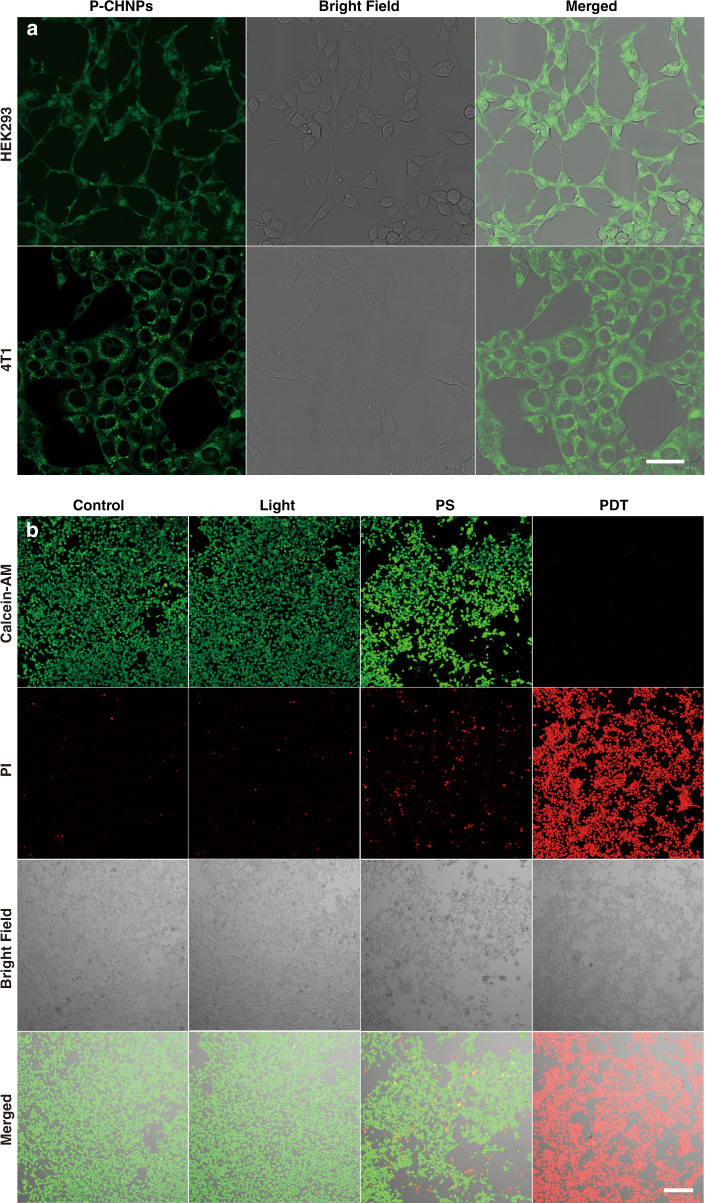
Fluorescence imaging with P-CHNPs and in vitro anticancer effect of P-CHNPs. **a** Confocal fluorescence images of HEK293 and 4T1 cells treated with the P-CHNPs. [P-CHNPs] = 100 μg mL^−1^. Scale bar: 50 μm. **b** Optical images of Calcein-AM and PI co-stained 4T1 cells treated with nothing (Control), light irradiation (Light), P-CHNPs (PS), and both light irradiation and P-CHNPs (PDT). Light irradiation: 400–700 nm, 100 mW cm^−2^, 20 min. [P-CHNPs] = 200 μg mL^−1^. Calcein-AM (Ex: 488 nm; Em: 505–525 nm) and PI (Ex: 552 nm; Em: 605–625 nm), [Calcein-AM] = 2 μM, [PI] = 2 μM. Scale bar: 200 μm

To investigate the fluorescence imaging potential of P-CHNPs endowed by solvothermal reaction, HEK293 and 4T1 cells were incubated with P-CHNPs (100 μg mL^−1^) for 12 h. After being washed with PBS three times, the P-CHNPs stained cells were prepared for fluorescence imaging. As shown in Fig. 4a, strong fluorescence signals were observed, enabling imaging-guided PDT. Considering the excellent ROS (^1^O_2_ and •OH) generation capability, P-CHNPs were incubated with 4T1 cells to analyze the in vitro cancer cell ablation efficiency. To optimize the light dose for PDT, 4T1 cells were irradiated with light (400–700 nm, 100 mW cm^−2^) for 5, 10, 20, 30 min after incubated with/out P-CHNPs (200 μg mL^−1^) for 12 h, and then subsequently stained with the calcein-acetoxymethyl ester (calcein-AM) and propidium iodide (PI) for cell viability analysis via CLSM. As shown in Fig. S14, with an increment of light irradiation time, more cells were PI-positive. After 10 min light irradiation, about half of the 4T1 cells were PI-positive, once the light irradiation time reached 20 min, all the cells were ablated, ensuring excellent in vitro PDT effect. Meanwhile, the 4T1 cells only treated with light irradiation i.e., the light group showed the calcein-AM fluorescence, indicating that light irradiation alone did not induce cellular phototoxicity. To further study the cellular response to P-CHNPs, 4T1 cells treated with nothing (i.e., control group), light irradiation (i.e., Light group), P-CHNPs (i.e., PS group), and both of Light irradiation and P-CHNPs (i.e., PDT group) were analyzed and compared as shown in Fig. 4b. The 4T1 cells only treated with light irradiation showed the calcein-AM fluorescence, indicating that light irradiation alone did not induce cellular phototoxicity. And part of 4T1 cells treated with P-CHNPs were propidium iodide (PI) positive. It could be deduced that part of 4T1 cells were ablated by •OH generated within intracellular response process, which was consistent with CCK-8 results as shown in Fig. 3a. Meanwhile, all 4T1 cells treated with both P-CHNPs and light irradiation were PI-positive, indicating the excellent in vitro PDT effect. To investigate the generalizability of P-CHNPs for treating different cancers, various types of cancer cell lines, including HeLa, PC3, and SCC7 cells, were incubated with/out P-CHNPs and then treated with light irradiation (Fig. S15). All cells treated with both P-CHNPs and light irradiation were PI-positive, demonstrating the generality of P-CHNPs based PDT. Meanwhile, all cells treated with only light irradiation showed the obvious calcein-AM fluorescence, indicating that light irradiation alone did not induce cellular phototoxicity and eliminating any interference from light irradiation.

#### In vivo photodynamic anticancer effect of P-CHNPs

**Fig. 5:**
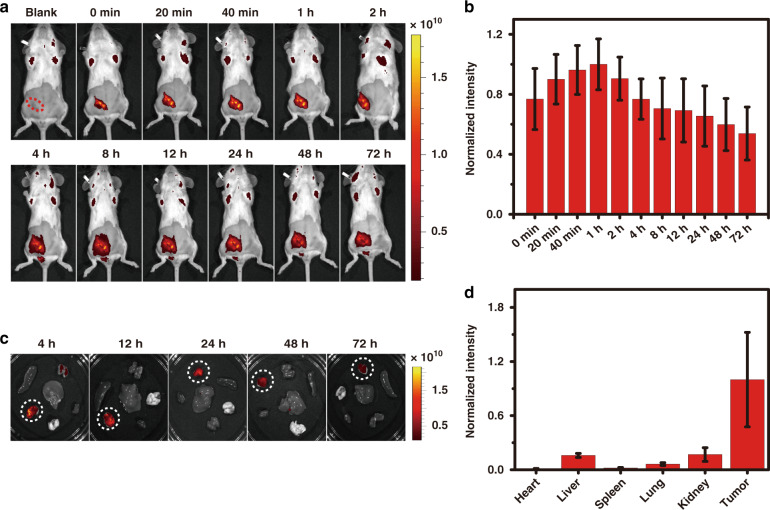
In vivo biodistribution of P-CHNPs. **a** Time-sequenced in vivo fluorescence images of P-CHNPs treated mice (8 mg kg^−1^). The circle indicated the tumor site. **b** Normalized fluorescence intensity in tumor sites at different monitoring times post-injection of P-CHNPs. Error bars were based on standard deviations (*n* = 4). **c** Time-sequenced ex vivo fluorescence images of tumors and major organs excised from P-CHNPs treated mice (8 mg kg^−1^). The circle indicated the tumor tissue. **d** Normalized fluorescence intensity in tumor and major organs at 72 h post-injection of P-CHNPs. Error bars were based on standard deviations (*n* = 4)

Before performing the in vivo anticancer studies, the in vivo and ex vivo fluorescence images were acquired at various time intervals to study the biodistribution of the administrated P-CHNPs (Fig. 5). To dynamically monitor the distribution of administrated P-CHNPs in the tumor site, the time-sequenced in vivo fluorescence images of P-CHNPs treated mice were recorded and analyzed (Fig. 5a, b). Once intratumorally injected of P-CHNPs, its strong fluorescence was observed in the tumor site, and the fluorescence signal covered the whole tumor site and reached the maximum at 1 h, which indicated that the P-CHNPs could efficiently reach the whole tumor, making it the optimal time point for the PDT applications. With a further increment of time, the fluorescence signal of P-CHNPs in the tumor site was gradually weakened, which could be attributed to the diffusion of P-CHNPs to the surroundings. Even so, the fluorescence signal of P-CHNPs in the tumor site were still apparent at 72 h post-injection, demonstrating the relative strong retention ability of P-CHNPs in the tumor site. The ex vivo fluorescence images of major organs and tumors excised from mice at 4, 12, 24, 48, and 72 h post intratumoral injection of P-CHNPs (8 mg kg^−1^) were also captured for analysis (Fig. 5c, d), the tumor showed much stronger fluorescence signal than other organs within 72 h post-injection of P-CHNPs, which was consistent with the in vivo fluorescence images of P-CHNPs treated mice. All these results demonstrated the effective preferential accumulation of P-CHNPs in tumors.

**Fig. 6:**
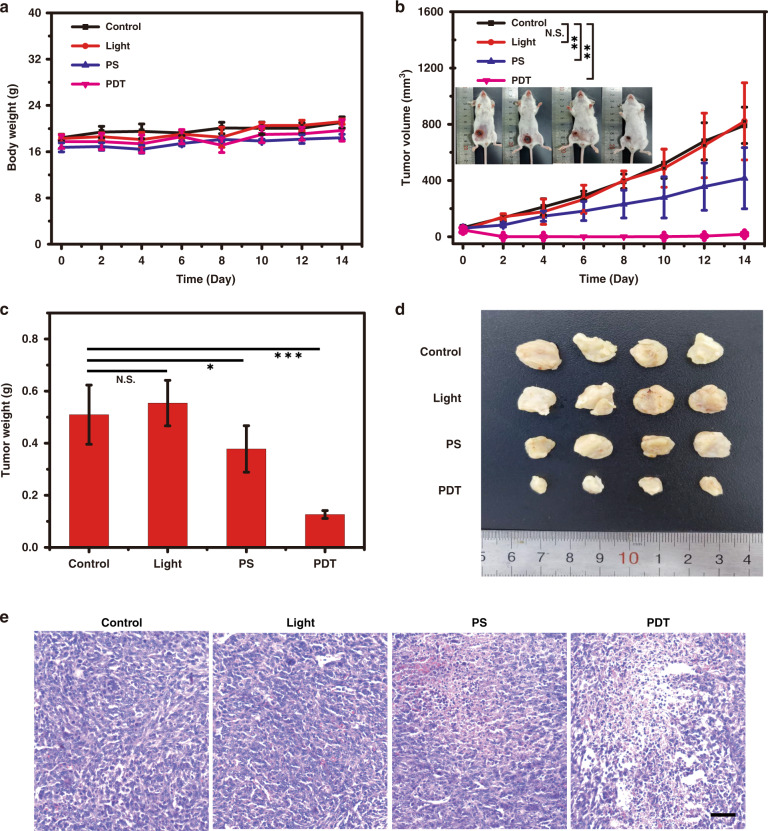
In vivo PDT efficiency of P-CHNPs. **a** Body weights of 4T1-tumor-bearing mice in all groups (*n* = 4). **b** Tumor growth curves of 4T1-tumor-bearing mice in different groups. Inset photographs recorded mice at Day 14, from left to right: Control, Light, PS and PDT. Error bars were based on standard deviations (*n* = 4). **c** Statistical analysis of tumor weight on day 14 posttreatment (*n* = 4). **d** The image of excised tumors on day 14 posttreatment. **e** Histological studies (H&E) of tumors from different groups. N.S. no significant difference, *P < 0.05, **P < 0.01, ***P < 0.001, *n* = 4. Scale bar: 200 μm

The synthesized P-CHNPs have shown excellent oxidative stress amplification capability, good biocompatibility, and excellent in vitro PDT effect, making P-CHNPs a promising in vivo PDT agent. The orthotopic breast tumor model, which is more similar to the practical clinical pathogenesis, was established by inoculating 4T1 cells in the abdominal mammary fat pad. Forty minutes after intratumorally injection of P-CHNPs (8 mg kg^−1^), mice were immediately irradiated with light (400–700 nm, 100 mW cm^−2^) for 20 min for the PDT group or 0 min for the PS group, respectively. While the mice in Control and Light groups were only treated with PBS or light irradiation, respectively. Moreover, the temperature during treatment was monitored by an IR thermal camera (Fig. S16), with an increment of treatment time, the temperature at the tumor site in PDT groups rose slowly. After 20 min irradiation, the temperature reached 37.5 °C, which was lower than the temperature required for photothermal therapy (PTT). Thus, PTT-induced interference could be efficiently eliminated from this treatment. After treatment, the mice weight and tumor size were measured every 2 days, there was no significant body weight loss for all groups within 2 weeks as shown in Fig. 6a. It was obvious that the tumor size of mice in Light and Control groups kept increasing quickly, while the tumor size increment rate of PS treated mice was significantly lowered as compared with that of Light and Control groups, indicating the efficient oxidative stress regulation in TME due •OH generated via Fenton reaction under the assistant of P-CHNPs (Fig. 6b and Fig. S17). Moreover, the tumor size of PDT treated mice was significantly decreased, which was comparable or better than common PDT as shown in Table S1. The boosted PDT with 97.7% tumor inhibition rate could be attributed to the amplified oxidative stress by P-CHNPs under light irradiation, wherein hypoxia was relieved, and GSH was depleted, thus the high quantity of ROS (^1^O_2_ and •OH) was generated in the tumor site. Another identical independent experiment was carried out, and a similar boosted PDT phenomenon was observed (Fig. S18), confirming the reproducibility of the excellent therapeutical outcomes endowed by the proposed PDT strategy.

All mice were sacrificed on day 14, the practical tumor weight and tumor morphology were shown in Fig. 6c, d. The tumors of the PS group were smaller than that of the Light and Control groups, indicating that the efficient generation of cytotoxic •OH. Moreover, the tumors of the PDT group were significantly decreased, demonstrating the light amplified oxidative stress in TME and consequently boosted PDT. It was worth to note that conventional PDT could only suppress the tumor growth, the amplified oxidative stress in TME remarkably improved the therapeutic outcomes. The tumors were also harvested for hematoxylin and eosin (H&E) staining to observe the histological changes. As shown in Fig. 6e, no obvious abnormalities and lesions could be observed in the tumor slices stained with H&E for mice without any treatment or with light irradiation. Limited and noticeable nucleus dissociation and necrosis were found for the tumor slices in PS and PDT groups respectively, indicating cell death within the tumors.

In order to further evaluate the performance of P-CHNPs as a promising PDT agent, one commercial photosensitizer of Ce6 was selected for comparative studies. Various concentrations of Ce6 were incubated with normal cells and cancer cells for different incubation times respectively, and the cell viability was evaluated using CCK-8. There was no significant difference between the control cells and normal/cancer cells treated with Ce6 in the concentration of 0.25–3.5 μM (Fig. S19), indicating its negligible dark toxicity and excellent biocompatibility. While P-CHNPs exhibited remarkable toxicity to cancer cells but not to normal cells due to efficient •OH generation in cancer cells via decomposition of the endogenous H_2_O_2_ under mild acidity condition (Fig. 3a). To analyze the in vitro cancer cell ablation efficiency of Ce6, 4T1 cells were incubated with/out Ce6 (3.0 μM). After light irradiation (400–700 nm, 100 mW cm^−2^, 20 min), the cells were stained with calcein-AM and PI for viability analysis using CLSM. As shown in Fig. S20, all cells treated with both Ce6 and light irradiation, i.e., PDT group were PI-positive, since PI is a fluorescent nucleic acid stain that can permeate only the damaged membranes, it can be deduced that membranes of the nucleus were damaged by PDT effect, which was also observed in P-CHNPs based PDT effect. Before performing the in vivo studies, the biodistribution of administrated Ce6 was analyzed using in vivo and ex vivo fluorescence images. After intratumoral injection of Ce6 (5 mg kg^−1^, typical clinical dosage), time-sequenced in vivo fluorescence images of Ce6 treated mice and ex vivo fluorescence images of tumors and major organs excised from Ce6 treated mice were recorded (Fig. S21). Ce6 accumulated in the tumor site at first, then quickly diffused to the liver, indicating that the Ce6 could be metabolized quickly by liver^52^. The short retention time limited the therapeutical outcomes. While the P-CHNPs possessed a longer retention time (Fig. 5), which ensured efficient generation of •OH and O_2_ as well as GSH depletion, amplifying oxidative stress in TME and consequently boosting therapeutical outcomes. Then, Ce6-based (5 mg kg^−1^) and P-CHNPs-based (8 mg kg^−1^) in vivo photodynamic anticancer therapy were performed at similar conditions (Fig. S18). The tumor size of mice in Control (treated with PBS), Light (treated with light irradiation), Ce6 (treated with Ce6) groups kept increasing quickly, while the tumor size increment rate of P-CHNPs treated mice was significantly lowered. It confirmed that Ce6 had negligible dark toxicity and P-CHNPs possessed efficient oxidative stress regulation in TME. Meanwhile, the tumor size and weight of mice in Ce6 PDT (treated with both Ce6 and light irradiation) and P-CHNPs PDT (treated with both P-CHNPs and light irradiation) groups were significantly decreased, demonstrating the superior anticancer effect. Considering that P-CHNPs had much lower ^1^O_2_ generation capability than Ce6 but similar therapeutical outcomes, the excellent anticancer effect of P-CHNPs could be attributed to the efficient oxidative stress regulation in TME. In order to further boost its anticancer effect, the P-CHNPs could be optimized, e.g. improve ^1^O_2_ generation capability, shift the absorption band to the longer wavelength to increase the penetration depth and subsequently ensure PDT effect exists in the whole tumor, functionalize the nanoparticles to prolong the retention time and further amply the oxidative stress in TME, etc.

### In vivo biocompatibility of P-CHNPs

In addition, the in vivo biocompatibility is another key parameter to evaluate the PDT agent. The blood was collected for routine hematology assays and blood biochemistry analysis on days 1, 8, and 14 after administration of P-CHNPs (8 mg kg^−1^) via subcutaneous injection. As shown in Fig. S22 and Table S2, no significant abnormities in hematology parameters and biochemical indicators, related to the hepatic function and kidney function, were observed, indicating the synthesized P-CHNPs have no obvious in vivo toxicity. Meanwhile, the major organs including heart, kidney, liver, lung, and spleen were also collected from sacrificed mice, which were treated with PDT, PS, etc., for analysis via H&E staining. As shown in Fig. S23, there were no signs of organ lesions for all groups, confirming the excellent in vivo biocompatibility of P-CHNPs.

## Discussion

Hemin, Fe (III)-contained protoporphyrin IX, could be endogenously produced in the human body, ensuring superior biocompatibility for its biological applications. Although hemin inherently possesses catalyzation capability, its efficiency for oxidative stress amplification in TME and anticancer effect is extremely limited. Facile solvothermal carbonization reaction made hemin lose the iron to form deferrization porphyrin fragments or carbonized products with ^1^O_2_ generation capability, and the O, N-containing groups combined with the released Fe ion stemming from hemin deferrization, enabling the formed CHNPs to act as photosensitizer with oxidative stress amplification capability in TME. Encapsulating CHNPs with polymer to form P-CHNPs not only maintained the spectral characteristics but also improved the biocompatibility. Meanwhile, the formed P-CHNPs possessed brand-new and improved features including fluorescence emission, high ROS (including singlet oxygen and hydroxyl radicals) generation efficiency, oxygen self-supplement, GSH depletion, etc., enabling efficient oxidative stress amplification in TME for boosting photodynamic anticancer therapy. Moreover, the oxidative stress regulation in TME selectively occurred in tumor site featured with H_2_O_2_ overexpression (0.1–1 mM), so •OH is only generated in the tumor-specific microenvironment but does little harm to normal tissues. Considering the increased glucose metabolic rates in tumors, glucose oxidase could be applied to catalyze glucose to generate H_2_O_2_ and subsequently amply the oxidative stress. Furthermore, the oxidative stress could be further amplified in the PDT process once light irradiation was employed via a photo-Fenton reaction. All these features made P-CHNPs an excellent PS for photodynamic anticancer therapy. The in vivo tumor ablations experimental results showed that P-CHNPs based PDT could reduce the tumor size instead of suppressing tumor growth, which was almost unachievable in common PDT. The excellent therapeutical outcomes could be attributed to (I) light irradiation triggered the Fe (III)/Fe (II) redox switch and thus the termination of oxidative stress regulation in TME via Fenton/Fenton-like reactions was avoided, (II) high generation efficiency of •OH and O_2_ due to P-CHNPs enabled TME regulation and light amplified oxidative stress, (III) O_2_ self-supplied in TME which subsequently relieved oxygen deficiency and improved the generation of ^1^O_2_, (IV) oxidative stress-induced damage to the tumor was exacerbated by GSH depletion. Moreover, superior in vitro and in vivo biocompatibility and PDT effect make the P-CHNPs a great potential therapeutic agent for future translational research.

## Materials and methods

### Synthesis of CHNPs

The CHNPs were synthesized using the common solvothermal reaction. Briefly, 15 mg of hemin was dispersed in 25 mL of ethyl alcohol, which was followed by ultrasonication for 15 min and subsequently heating at 240 °C for 24 h in an autoclave. After being air-cooled down to room temperature, the products were filtered by 0.22 μm membranes and purified with centrifugal ultrafiltration three times, finally, the CHNPs were obtained.

### Encapsulation of CHNPs with polymer

To further improve the biocompatibility, the CHNPs were encapsulated with amphiphilic polymer. DSPE-mPEG (2 mg) and CHNPs (1 mg) were dissolved in 2 mL of tetrahydrofuran (THF), then the mixture was quickly injected into 18 mL of water under continuous sonication for 2 min. After the THF was evaporated, the aqueous solution was filtered with 0.22 μm membranes and purified with centrifugal ultrafiltration. Finally, the P-CHNPs were obtained and kept at 4 °C for further use.

### Singlet oxygen detection

The ^1^O_2_ generation efficiency of P-CHNPs was assessed by ABDA, a ^1^O_2_ indicator. Briefly, 50 μM ABDA and 50 μg mL^−1^ P-CHNPs were mixed to 2 mL aqueous solution. Subsequently, the cuvette was exposed to light (400–700 nm, 70 mW cm^−2^) for different time intervals. Corresponding absorption spectra were recorded immediately after each irradiation. In the presence of ^1^O_2_, the absorbance of ABDA would significantly decrease due to the oxidative decomposition effect of ^1^O_2_ on ABDA.

### Hydroxyl radicals detection

To evaluate the •OH generation capability of P-CHNPs, TMB, which can be oxidized by the highly reactive •OH to possess a maximum absorbance at about 650 nm, was selected as the •OH indicator. The PBS buffer solution (pH 6.5) containing TMB (800 μM) was mixed with P-CHNPs (50 μg mL^−1^) in the presence of H_2_O_2_ (300 μM) with or without light irradiation. The TMB solutions treated with P-CHNPs or H_2_O_2_ alone with/without light irradiation were used as control. The absorption spectra of the mixture solution were recorded.

Considering that the •OH generation capability by P-CHNPs was pH-dependent, P-CHNPs (50 μg mL^−1^), H_2_O_2_ (300 μM), and TMB (800 μM) were added to PBS buffer with different pH values (pH 4.5, 5.0, 5.5, 6.0, 6.5,7.0, and 7.4). After reaction for 60 min at 37 °C, the absorption spectra of the mixture solution were recorded.

### The depletion of GSH

The GSH solution (650 μM), P-CHNPs aqueous solution (100, 200 μg mL^−1^), and extraction buffer solution were added sequentially. The mixture solution was maintained at 37 °C for 60 min, then centrifuged to remove P-CHNPs, and finally, the supernatant was collected for further assay. The GSH depletion was measured according to the Kit assay protocol.

### Cellular toxicity tests

4T1 cells and L02 cells were cultivated in RPMI 1640 (Roswell Park Memorial Institute) supplied with 10% fetal bovine serum (FBS), HEK293 cells, and HeLa cells were cultivated in DMEM (Dulbecco’s modified eagle medium) supplied with 10% FBS. Cultures were maintained at 37 °C under a humidified atmosphere containing 5% CO_2_. Cells were plated into a 96-well plate at a density of 5000 cells per well and cultured for 24 h, respectively. Then the medium was replaced by 100 μL of fresh culture medium containing P-CHNPs with various concentrations (50–300 μg mL^−1^). The medium and untreated cells were used as blank and control, respectively. After 24 or 48 h, the cultured medium was removed, and the cells were washed three times with PBS to remove the residual P-CHNPs. 100 μL of fresh medium containing 10 μL of CCK-8 was added to each well. After 2 h incubation at 37 °C, the absorbance at 450 nm was measured using a microplate reader. The cells viability was calculated by the following equation:

$${\mathrm{Cell}}\,{\mathrm{Viability}}\,\left( {{{\mathrm{\% }}}} \right) = \frac{{{\mathrm{OD}} - {\mathrm{OD}}_{{{{\mathrm{Blank}}}}}}}{{{\mathrm{OD}}_{{{{\mathrm{Control}}}}} - {\mathrm{OD}}_{{{{\mathrm{Blank}}}}}}} \ast 100$$

### In vitro PDT effect evaluation

To evaluate the in vitro PDT effect of P-CHNPs, the 4T1 cells were seeded in a confocal dish at a density of 5000 cells per well, and then cultured at 37 °C, 5% CO_2_ atmosphere for 24 h. P-CHNPs (200 μg mL^−1^) were added to each well. After 12 h incubation, the P-CHNPs treated cells were washed with PBS three times to remove the residual P-CHNPs, which was followed by light irradiation (400–700 nm, 100 mW cm^−2^, 20 min). After light irradiation, cells were incubated with fresh media for 4 h. And then, a fresh FBS-free culture medium containing calcein-AM (2 μM) and PI (2 μM) were added and incubated for another 30 min. After washing three times to remove the residual calcein-AM and PI, the treated cells were proceeded for fluorescent imaging using confocal laser scanning microscopy.

### Animal model

All the animal experiments were approved by the bioethics committee of the school of biomedical engineering, Shanghai Jiao Tong University, and were consistent with regulations for the care and use of experimental animals in China. Five weeks old female BALB/c mice, originally purchased from SPF experimental animal center, were used to establish tumor model. Briefly, 1 × 10^6^ 4T1 cells were injected into the breast fat pad of each female BALB/c mouse (*n* = 4) to establish the orthotopic breast tumor model. Once the tumors grew to about 50 mm^3^ in volume, the in vivo experiments were performed.

### Therapeutic evaluation in tumor-bearing mice

The mice were randomly divided into four groups. Control group: administration with PBS (40 μL) alone; Light group: administration with PBS (40 μL) and light irradiation (400–700 nm, 100 mW cm^−2^, 20 min); PS group: administration with P-CHNPs (8 mg kg^−1^, 40 μL); PDT group: administration with P-CHNPs (8 mg kg^−1^, 40 μL) and light irradiation (400–700 nm, 100 mW cm^−2^, 20 min). P-CHNPs or PBS were injected intratumorally. During the treatment period (14 days), the tumor volume of all mice was measured every other day using a vernier caliper. On day 14, mice were sacrificed, then tumors in all groups were harvested and weighed. For histological analysis, the hematoxylin-eosin (H&E) staining of tumor slices was carried out. The greatest longitudinal diameter (length) and the greatest transverse diameter (width) were used to calculate the tumor volume:

$${\mathrm{Tumor}}\,{\mathrm{Volume}} = {\mathrm{Width}} \ast {\mathrm{Width}} \ast {\mathrm{Length}}/2$$

The tumor growth inhibition rates (*IR*) were calculated via the following formula:

$${\mathrm{IR}}\left( {{{\mathrm{\% }}}} \right) = \left( {1 - \frac{{{\mathrm{TV}}_t}}{{{\mathrm{TV}}_c}}} \right) \ast 100$$

where *TV*t represents the mean tumor volume of the treated groups and *TV*c represents the mean tumor volume of the control group.

### Statistical analysis

Data reported in this work were presented as the mean ± standard deviation (s.d.). Statistical analysis of data were performed with *T*-test analysis of variance. The level of significance was defined as *P < 0.05, **P < 0.01, ***P < 0.001, ****P < 0.0001.

## Supplementary information

Supporting Information

## References

[1] Wild, C. P., Weiderpass, E. & Stewart, B. W.*World Cancer Report: Cancer Research for Cancer Prevention* (International Agency for Research on Cancer, 2020). 39432694

[2] Yin F (2017). Functionalized 2D nanomaterials for gene delivery applications. Coord. Chem. Rev..

[3] Ding SH (2021). Near-infrared light excited photodynamic anticancer therapy based on UCNP@AIEgen nanocomposite. Nanoscale Adv..

[4] Pang W (2020). Nucleolus-targeted photodynamic anticancer therapy using renal-clearable carbon dots. Adv. Healthc. Mater..

[5] Dougherty TJ (1998). Photodynamic therapy. J. Natl Cancer Inst..

[6] Li XS (2020). Clinical development and potential of photothermal and photodynamic therapies for cancer. Nat. Rev. Clin. Oncol..

[7] Xie ZJ (2020). Black phosphorus-based photothermal therapy with aCD47-mediated immune checkpoint blockade for enhanced cancer immunotherapy. Light Sci. Appl..

[8] Bao X (2018). In vivo theranostics with near-infrared-emitting carbon dots-highly efficient photothermal therapy based on passive targeting after intravenous administration. Light Sci. Appl..

[9] Agostinis P (2011). Photodynamic therapy of cancer: an update. Cancer J. Clin..

[10] Li BH (2016). Photosensitized singlet oxygen generation and detection: recent advances and future perspectives in cancer photodynamic therapy. J. Biophotonics.

[11] Lin LS (2020). Singlet oxygen luminescence image in blood vessels during vascular-targeted photodynamic therapy. Photochem. Photobiol..

[12] Dolmans DEJGJ, Fukumura D, Jain RK (2003). Photodynamic therapy for cancer. Nat. Rev. Cancer.

[13] Gu BB, Yong KT, Liu B (2018). Strategies to overcome the limitations of AIEgens in biomedical applications. Small Methods.

[14] Gu BB (2017). Precise two-photon photodynamic therapy using an efficient photosensitizer with aggregation-induced emission characteristics. Adv. Mater..

[15] Markovic ZM (2012). Graphene quantum dots as autophagy-inducing photodynamic agents. Biomaterials.

[16] Li Z (2020). NIR/ROS-responsive black phosphorus QD vesicles as immunoadjuvant carrier for specific cancer photodynamic immunotherapy. Adv. Funct. Mater..

[17] Chen S (2020). Carbon dots based nanoscale covalent organic frameworks for photodynamic therapy. Adv. Funct. Mater..

[18] Ge JC (2014). A graphene quantum dot photodynamic therapy agent with high singlet oxygen generation. Nat. Commun..

[19] Fan WP, Huang P, Chen XY (2016). Overcoming the Achilles’ heel of photodynamic therapy. Chem. Soc. Rev..

[20] Liang JH (2020). A tailored multifunctional anticancer nanodelivery system for ruthenium-based photosensitizers: tumor microenvironment adaption and remodeling. Adv. Sci..

[21] Jiang S (2020). Synergistic anticancer therapy by ovalbumin encapsulation-enabled tandem reactive oxygen species generation. Angew. Chem. Int. Ed..

[22] Liu CH (2020). An open source and reduce expenditure ROS generation strategy for chemodynamic/photodynamic synergistic therapy. Nat. Commun..

[23] Liu Y (2018). All-in-one theranostic nanoagent with enhanced reactive oxygen species generation and modulating tumor microenvironment ability for effective tumor eradication. ACS Nano.

[24] Tang ZM (2019). Chemodynamic therapy: tumour microenvironment-mediated Fenton and Fenton-like reactions. Angew. Chem. Int. Ed..

[25] Li TL (2019). Photo-Fenton-like metal-protein self-assemblies as multifunctional tumor theranostic agent. Adv. Healthc. Mater..

[26] Zhang C (2017). An O_2_ self-supplementing and reactive-oxygen-species-circulating amplified nanoplatform via H_2_O/H_2_O_2_ splitting for tumor imaging and photodynamic therapy. Adv. Funct. Mater..

[27] Wang M (2020). Au_2_Pt-PEG-Ce6 nanoformulation with dual nanozyme activities for synergistic chemodynamic therapy/phototherapy. Biomaterials.

[28] Feng LL (2017). Multifunctional UCNPs@MnSiO_3_@g-C_3_N_4_ nanoplatform: improved ROS generation and reduced glutathione levels for highly efficient photodynamic therapy. Biomater. Sci..

[29] Ju EG (2016). Copper(II)-graphitic carbon nitride triggered synergy: improved ROS generation and reduced glutathione levels for enhanced photodynamic therapy. Angew. Chem. Int. Ed..

[30] Kim J (2017). Continuous O_2_-evolving MnFe_2_O_4_ nanoparticle-anchored mesoporous silica nanoparticles for efficient photodynamic therapy in hypoxic cancer. J. Am. Chem. Soc..

[31] Panwar N (2019). Nanocarbons for biology and medicine: sensing, imaging, and drug delivery. Chem. Rev..

[32] Wu Q (2019). MnO_2_-laden black phosphorus for MRI-guided synergistic PDT, PTT, and chemotherapy. Matter.

[33] Ding YB, Zhu WH, Xie YS (2017). Development of ion chemosensors based on porphyrin analogues. Chem. Rev..

[34] Luo FQ (2015). Encapsulation of hemin in metal-organic frameworks for catalyzing the chemiluminescence reaction of the H_2_O_2_-luminol system and detecting glucose in the neutral condition. ACS Appl. Mater. Interfaces.

[35] Xue T (2012). Graphene-supported hemin as a highly active biomimetic oxidation catalyst. Angew. Chem. Int. Ed..

[36] Wang QG (2008). High catalytic activities of artificial peroxidases based on supramolecular hydrogels that contain heme models. Chemistry.

[37] Zhu SJ (2015). The photoluminescence mechanism in carbon dots (graphene quantum dots, carbon nanodots, and polymer dots): current state and future perspective. Nano Res..

[38] He LC (2020). Solvent-assisted self-assembly of a metal-organic framework based biocatalyst for cascade reaction driven photodynamic therapy. J. Am. Chem. Soc..

[39] Bai J (2018). A facile ion-doping strategy to regulate tumor microenvironments for enhanced multimodal tumor theranostics. J. Am. Chem. Soc..

[40] Gong T (2020). Full-process radiosensitization based on nanoscale metal-organic frameworks. ACS Nano.

[41] Bai S (2020). Ultrasmall iron-doped titanium oxide nanodots for enhanced sonodynamic and chemodynamic cancer therapy. ACS Nano.

[42] Zheng XT, Ananthanarayanan A, Luo KQ, Chen P (2015). Glowing graphene quantum dots and carbon dots: properties, syntheses, and biological applications. Small.

[43] Hola K (2014). Carbon dots-emerging light emitters for bioimaging, cancer therapy and optoelectronics. Nano Today.

[44] Wang Y (2014). Thickness-dependent full-color emission tunability in a flexible carbon dot ionogel. J. Phys. Chem. Lett..

[45] Drössler P (2003). Fluoresence quenching of riboflavin in aqueous solution by methionin and cystein. Chem. Phys..

[46] Devi LG, Nithya PM (2018). Photocatalytic activity of Hemin (Fe(III) porphyrin) anchored BaTiO_3_ under the illumination of visible light: synergetic effects of photosensitization, photo-Fenton & photocatalysis processes. Inorg. Chem. Front..

[47] Jahan M, Bao QL, Loh KP (2012). Electrocatalytically active graphene-porphyrin MOF composite for oxygen reduction reaction. J. Am. Chem. Soc..

[48] Redmond RW, Gamlin JN (1999). A compilation of singlet oxygen yields from biologically relevant molecules. Photochem. Photobiol..

[49] Gao L (2014). Plasmon-mediated generation of reactive oxygen species from near-infrared light excited gold nanocages for photodynamic therapy in vitro. ACS Nano.

[50] Wang JP (2020). A porous Au@Rh bimetallic core-shell nanostructure as an H_2_O_2_-driven oxygenerator to alleviate tumor hypoxia for simultaneous bimodal imaging and enhanced photodynamic therapy. Adv. Mater..

[51] Ruppert G, Bauer R, Heisler G (1993). The photo-Fenton reaction-an effective photochemical wastewater treatment process. J. Photochem. Photobiol. A: Chem..

[52] Li WJ (2016). Smart hyaluronidase-actived theranostic micelles for dual-modal imaging guided photodynamic therapy. Biomaterials.

