# Supplementary material for: Light amplified oxidative stress in tumor microenvironment by carbonized hemin nanoparticles for boosting photodynamic anticancer therapy
Source: Light Sci Appl. 2022 Mar 1;11:47. doi: 10.1038/s41377-021-00704-5 (PMC8885839; doi:10.1038/s41377-021-00704-5)
Supplement: Supplementary file 1 — Supporting Information [file 41377_2021_704_MOESM1_ESM.docx]

Supporting Information for

**Light Amplified Oxidative Stress in Tumor Microenvironment by Carbonized Hemin Nanoparticles for Boosting Photodynamic Anticancer Therapy**

Liyun Lin^1^, Wen Pang^1^, Xinyan Jiang^1^, Shihui Ding^1^, Xunbin Wei^1,2,3,^*, Bobo Gu^1,^*

^1^Med-X Research Institute and School of Biomedical Engineering, Shanghai Jiao Tong University, Shanghai 200030, China

^2^Biomedical Engineering Department, Peking University, Beijing 100081, China

^3^Key Laboratory of Carcinogenesis and Translational Research (Ministry of Education/Beijing), Peking University Cancer Hospital & Institute, Beijing, 100142, China

*Corresponding Author: xwei@bjmu.edu.cn (X. Wei); bobogu@sjtu.edu.cn (B. Gu)

**Experimental methods and materials**

Reagents and instruments

All chemicals were used without further purification unless stated. Hemin was purchased from Sangon Biotech (Shanghai, China). Ethanol, tetrahydrofuran (THF) and 9,10-Anthracenediyl-bis(methylene)-dimalonic acid (ABDA), propidium iodide (PI), 2′,7′-dichlorofluorescein diacetate (DCFH-DA) were purchased from Sigma (United States). 1,2-distearoyl-sn-glycero-3-phosphoethanolamine-N-methoxy (polyethylene glycol)-2000 (DSPE-mPEG) was purchased from Laysan (United States). 3,3',5,5'-tetramethylbenzidine (TMB) was purchased from Solarbio (Beijing, China). [Ru(dpp)_3_]Cl_2_ (RDPP) was obtained from Aladdin (Beijing, China). Calcein acetoxymethyl ester (calcein-AM) was obtained from Biolegend (United States). Cell Counting Kit-8 (CCK-8), 5,5-dimethyl- 1-pyrroline-N-oxide (DMPO), and 2,2,6,6- tetramethylpiperidine (TEMP) was obtained from Dojindo Molecular Technologies (Japan). CheKine Reduced Glutathione (GSH) Colorimetric Assay Kit was purchased from Abbkine Scientific (United States). RPMI medium, DMEM medium, and phosphate buffered saline (PBS, 1×) were purchased from HyClone (United States). Fetal calf serum was purchased from ScienCell (United States). Deionized water (Millipore Milli-Q grade) with a resistivity of 18.2 MΩ was applied in all experiments.

Fluorescence spectra were recorded using F-2700 Fluorescence Spectrophotometer (PerkinElmer, United States). UV-Vis absorption spectra were measured using UV1901PC spectrophotometer (Aucy, China). X-ray photoelectron spectroscopy (XPS) was conducted on an AXIS UltraDLD electron spectrometer (Shimadzu-Kratos, Japan) using 150 W Al Kα radiation. Transmission electron microscopy (TEM) images was taken using the TALOS F200X microscope operating at 200 kV (FEI, United States). Fourier transform infrared (FT-IR) spectra were measured using IR/Nicolet 6700 (Thermo Fisher, United States). Dynamic light scattering (DLS) was measured by Zetasizer Nano ZSP (Malvern, United Kingdom). Electron spin resonance (ESR) analysis was carried out with an ESR spectrometer (ESR5000) at room temperature (Bruker, Germany). The absorbance at 450 nm of CCK-8 cell viability assay was recorded using SpectraMax M5 Microplate Reader (Molecular Devices, USA). Cellular imaging was operated by a confocal laser scanning microscopy (CLSM) (Leica SP8, Germany). In vivo/ex vivo fluorescence images were recorded using IVIS Lumina III (PerkinElmer, Germany).

**The Fluorescence Quantum Yield Measurements.** The fluorescence quantum yield was measured by using riboflavin (Φ_Riboflavin_ = 0.23 in DMSO) as the reference. The relative quantum yields were determined according the equation^1^:

$$\Phi_{F(x)}=(\frac{A_{s}}{A_{x}})(\frac{F_{x}}{F_{s}}){(\frac{n_{x}}{n_{s}})}^{2}\Phi_{F(s)}$$

where *Φ* is the fluorescence quantum yield, *A* is the measured UV-vis absorption spectra intensity, *F* is the measured fluorescence spectra integrated area. *n* is the refractive index of the solvents used. Subscripts s and *x* refer to the standard and the test sample, respectively.

**The ^1^O_2_ Quantum Yield Measurements.** ^1^O_2_ quantum yield was determined by using Chlorin e6 (Ce6, *Φ*_Ce6_ = 0.66 in aqueous media) as a standard. The quantum yield was calculated according to the following equation^2^:

$$\Phi=\Phi_{Ce6}*\frac{K_{P-CHNPs}*A_{Ce6}}{K_{Ce6}*A_{P-CHNPs}}$$

where Φ is the ^1^O_2_ quantum yield, *A* is the measured UV-vis absorption spectra integrated area, *K* is the slope of consumption rate linear equation of ABDA after different time exposure.

**Electron Spin Resonance Spectroscopic Measurements.** All the ESR measurements were performed by a Bruker ESR5000 spectrometer at ambient temperature. The sample was put into a quartz capillary tube and sealed using silicone grease. Then the capillary tube was inserted in the ESR cavity and the spectra were recorded. The spin trap DMPO was used to verify the generation of •OH. 600 μL of PBS buﬀer solution (pH 6.5) containing DMPO (7.5 μL) was mixed with P-CHNPs (50 μg mL^-1^) in the presence of H_2_O_2_ (600 μM) with or without light irradiation (400 - 700 nm, 100 mW cm^-2^, 20 min). The DMPO solutions treated with P-CHNPs alone with/without light irradiation or H_2_O_2_ with light irradiation were used as control. The spin trap TEMP was used to demonstrate the production of ^1^O_2_, 600 μL of PBS buﬀer solution (pH 6.5) contained TEMP (300 mM) was mixed with P-CHNPs (50 μg mL^-1^) with light irradiation (400 - 700 nm, 100 mW cm^-2^, 20 min). To further verified the ^1^O_2_ amplification resulted from catalyzing H_2_O_2_ to O_2_ by P-CHNPs, H_2_O_2_ (600 μM) was added to the reaction system. The TEMP solutions treated with P-CHNPs alone or H_2_O_2_ with light irradiation were used as control.

**In Vitro Cellular Oxygenation Detection.** The in vitro cellular hypoxia relief was confirmed by the confocal imaging based on the hypoxia-sensitive fluorescence probe Ru(dpp)_3_Cl_2_ (RDPP). 4T1 tumor cells were initially seeded into the confocal disk with a cell density of 10^5^ cells/disk. Cells were allowed to attach for 12 h, then medium was replaced and incubated with RDPP (10 μg mL^-1^) for 2 h. 1640 medium containing P-CHNPs (200 μg mL^-1^) was supplemented to replace the previous medium after rinsing with PBS. After 12 h incubation in dark, the P-CHNPs treated cells were washed with PBS for three times to remove the residual P-CHNPs, which positive group was followed by H_2_O_2_ (300 μM) treatment and incubated for another 30 min. For light irradiation groups, light was employed to irradiate the treated cells after co-incubation (400 - 700 nm, 100 mW cm^-2^, 20 min). The RDPP solutions treated with/without light irradiation were used as control. After washing three times with PBS, the treated cells were proceeded for imaging using confocal laser scanning microscopy.

**The Analysis of Intracellular ROS Generation.** DCFH-DA was employed as a ROS probe to evaluate P-CHNPs-induced intracellular oxidative stress. DCFH-DA could be rapidly hydrolyzed by intracellular esterase once internalized by cells and oxidized to fluorescent DCF by ROS. Briefly, the 4T1 cells were seeded in confocal dish at density of 5000 cells per well, which was followed by incubation at 37°C, 5% CO_2_ atmosphere for 24 h. Then, the 4T1 cells were incubated with various concentrations of P-CHNPs (0, 100, 200 μg mL^-1^) for 9 h. After washed with PBS for three times to remove the residual P-CHNPs, 4T1 cells were stained with DCFH-DA (10 μM) in FBS free medium. After 30 min incubation, the 4T1 cells were irradiated with light (400 - 700 nm, 200 mW cm^-2^) for different time, which was immediately followed by ﬂuorescence imaging by CLSM to analyze the intracellular ROS generation.

**Evaluation of** **Hemolysis.** Blood was obtained from the BALB/c mice. To isolate erythrocytes, the blood sample was centrifuged at 3,000 rpm for 10 min and washed five times with PBS solution. Then, 0.5 mL of 4% erythrocytes (v/v) was mixed with various concentrations of P-CHNPs solution (0.5 mL of 50, 100, 200, 400 μg mL^-1^), water and saline. After incubated for 12 h at 37℃，the corresponding mixture solutions were centrifuged at 4000 rpm for 10 min and the supernatants were collected. In order to assess the hemolytic activity, the released hemoglobin in supernatant was analyzed by monitoring the absorbance at 542 nm by using a microplate reader (Molecular Devices, SpectraMaxM5). The percentage of hemolysis acquired as follow:

$${(I}_{sample}-I_{0})/(I_{100}-I_{0})*100$$

where *I*_sample_, *I*_100_ and *I*_0_ refer to the absorbance of the erythrocytes with different concentrations P-CHNPs, the completely lysed red blood cells in distilled water, and no obvious hemolysis in PBS. All hemolysis assays were conducted with three replicates (n = 3).

**In Vivo/Ex Vivo Fluorescence Imaging:** At predetermined time point (such as 0, 20 min, 40 min, 1 h, 2 h, 4 h, 8 h, 12 h, 24 h, 48 h and 72 h) post-injection of photosensitizers, the mice were anesthetized for in vivo imaging. At 4, 12, 24, 48 and 72 h post-injection of photosensitzers, the tumors and major organs were excised from the treated mice for ex vivo imaging.

**In Vivo Systemic Toxicity Study.** In order to evaluate the systemic toxicity, the mice were divided into three groups. Control group: subcutaneous injection with PBS (40 μL); P-CHNPs group: administration with P-CHNPs (8 mg kg^-1^, 40 μL); PDT group: administration with P-CHNPs (8 mg kg^-1^, 40 μL) and light irradiation (400 - 700 nm, 100 mW cm^-2^, 20 min). In vivo blood biochemistry and blood routine analysis were comprehensively carried out to study the long-term biotoxicity of P-CHNPs. Blood from three groups were collected on day 1, 8, and 14. The hepatic function and kidney function parameters, including alkaline phosphatase (ALP), aspartate aminotransferase (AST), alanine transaminase (ALT), creatinine (CRE) and blood urea nitrogen (BUN), were monitored. For the blood routine analysis, indexes such as red blood cells (RBC), hematocrit (HCT), hemoglobin (HGB), platelets (PLT), mean platelet volume (MPV), mean corpuscular volume (MCV) and mean corpuscular hemoglobin concentration (MCHC) were evaluated.

**Supplementary results**

**Figure S1.** Representative TEM image of the CHNPs.


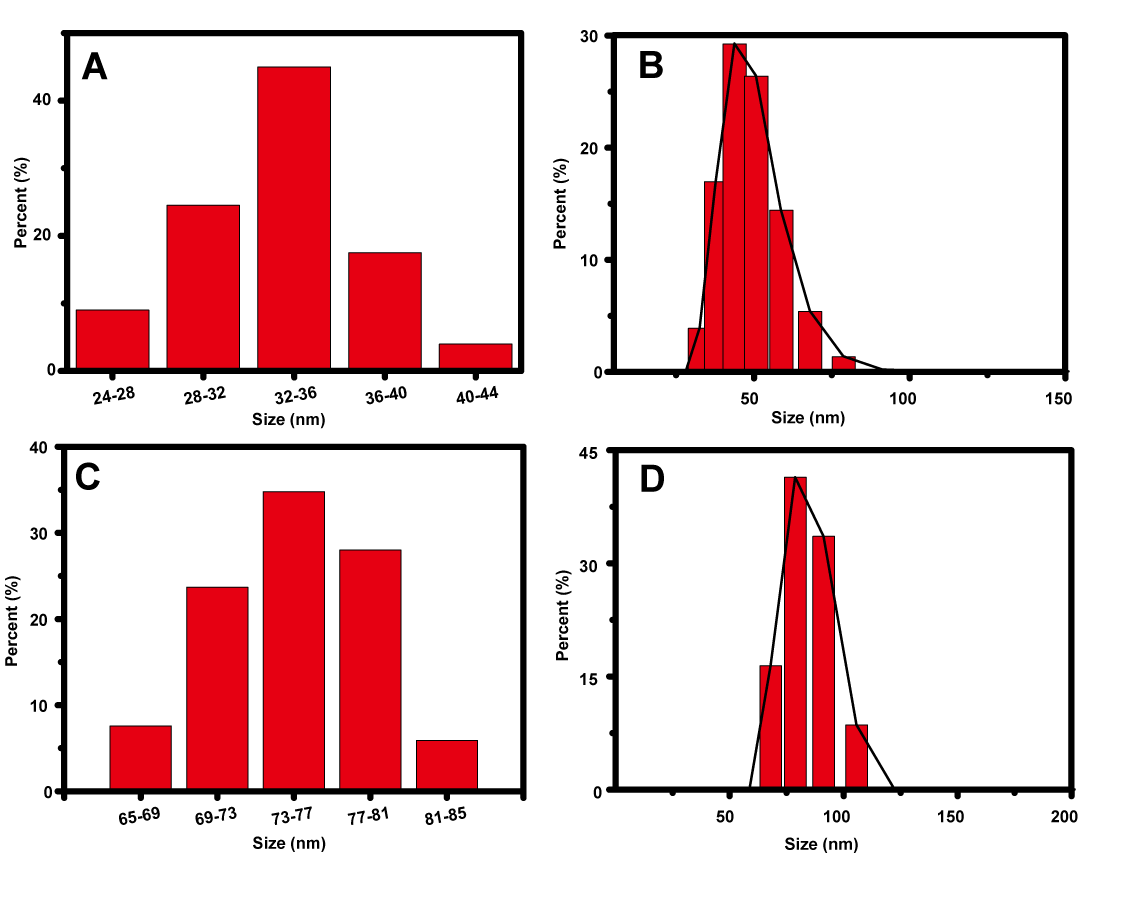


**Figure S2.** The TEM statistical cumulative particle size distributions of the (A) CHNPs and (C) P-CHNPs. The DLS results of (B) CHNPs and (D) P-CHNPs.


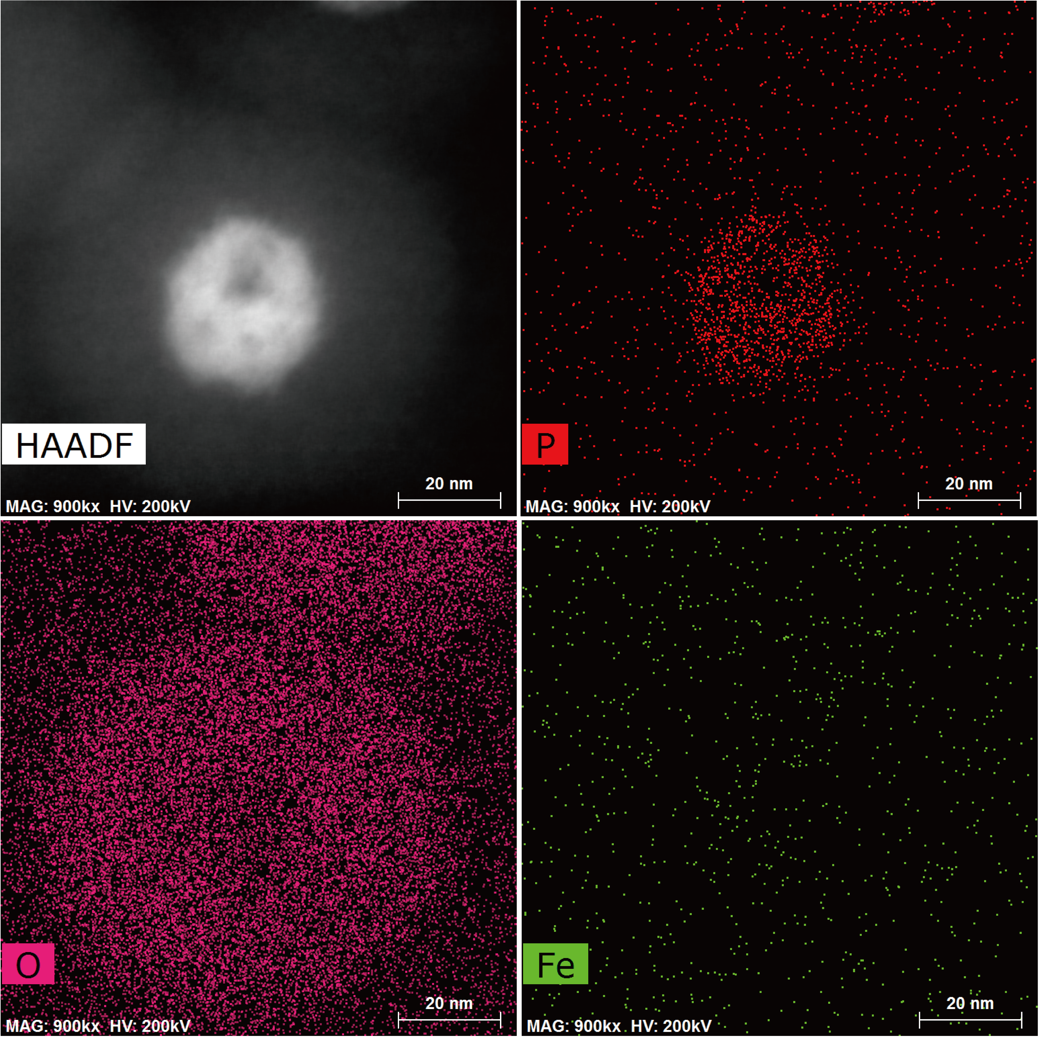


**Figure S3.** HAADF-STEM image and corresponding elemental distribution mapping of P, O, and Fe of the synthesized P-CHNPs.


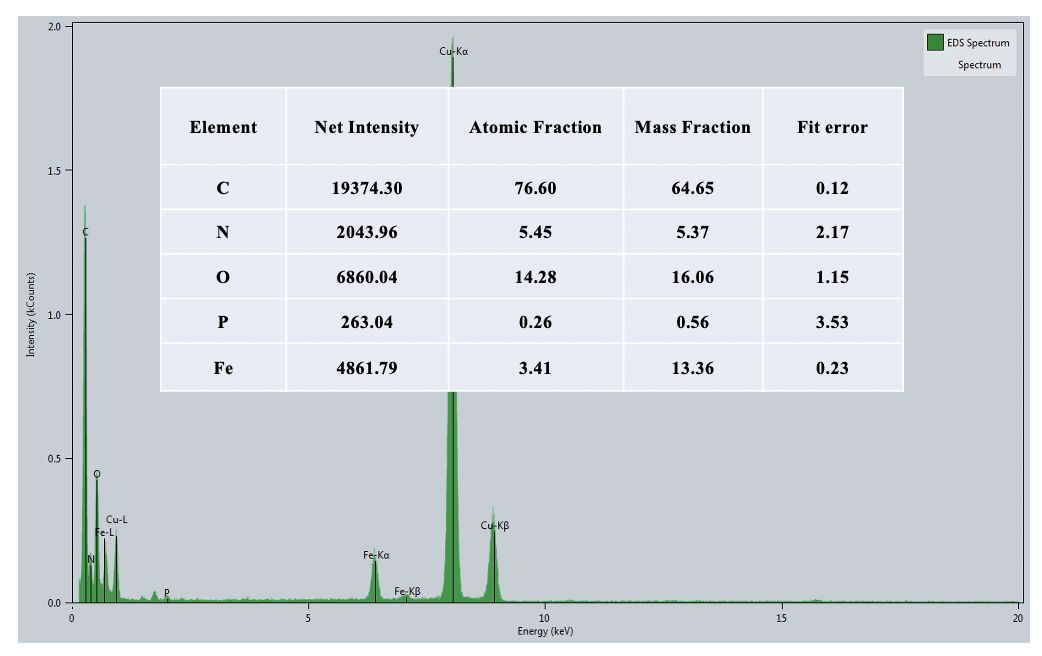


**Figure S4.** The corresponding EDX spectroscopy and elemental contents.


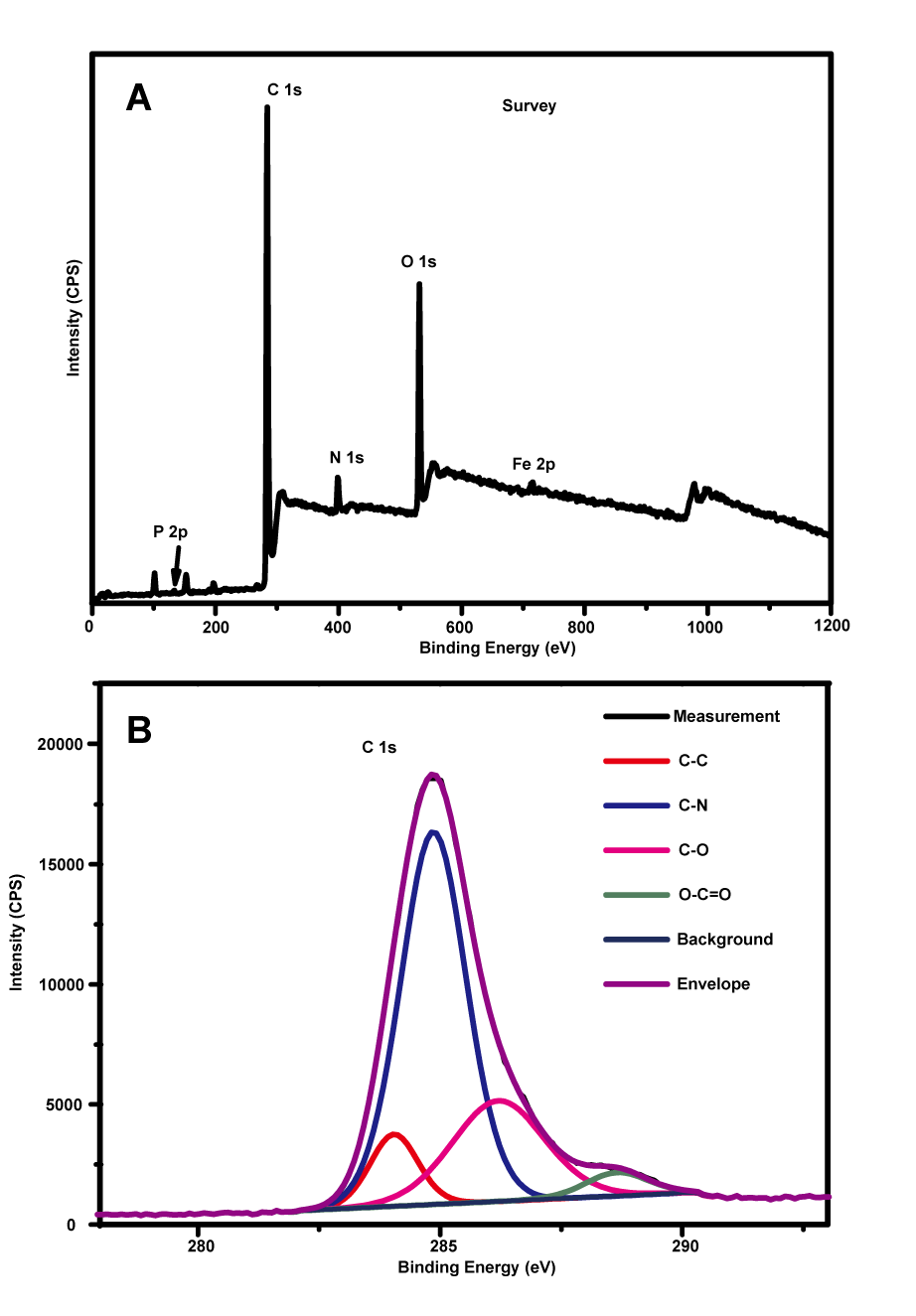


**Figure S5.** (A) The full-scan XPS and (B) high-resolution XPS of P-CHNPs for C 1s.


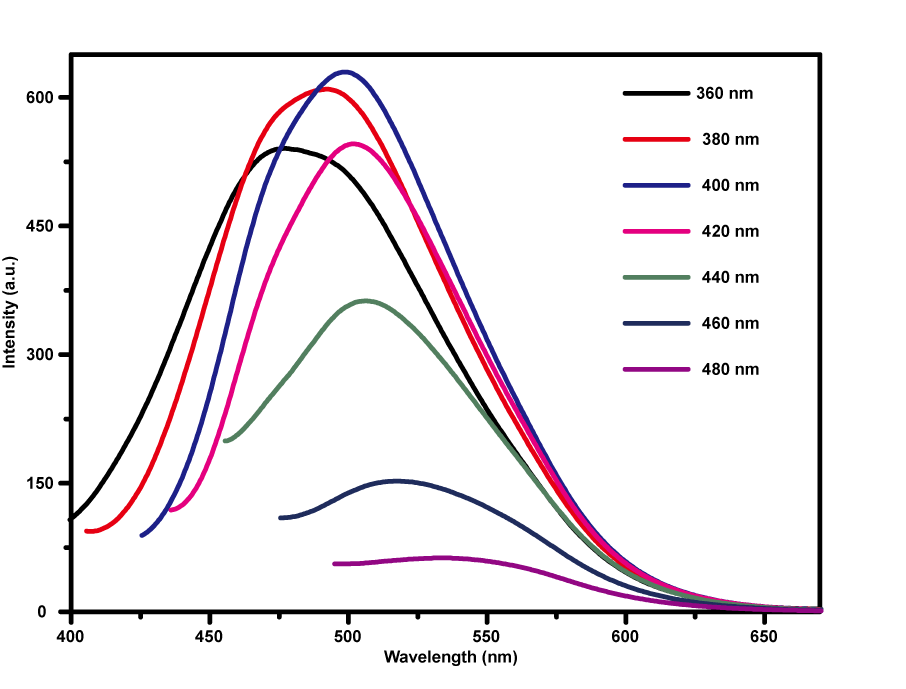


**Figure S6.** The fluorescence emission spectra of P-CHNPs under different excitation wavelengths. [P-CHNPs] = 25 μg mL^-1^.


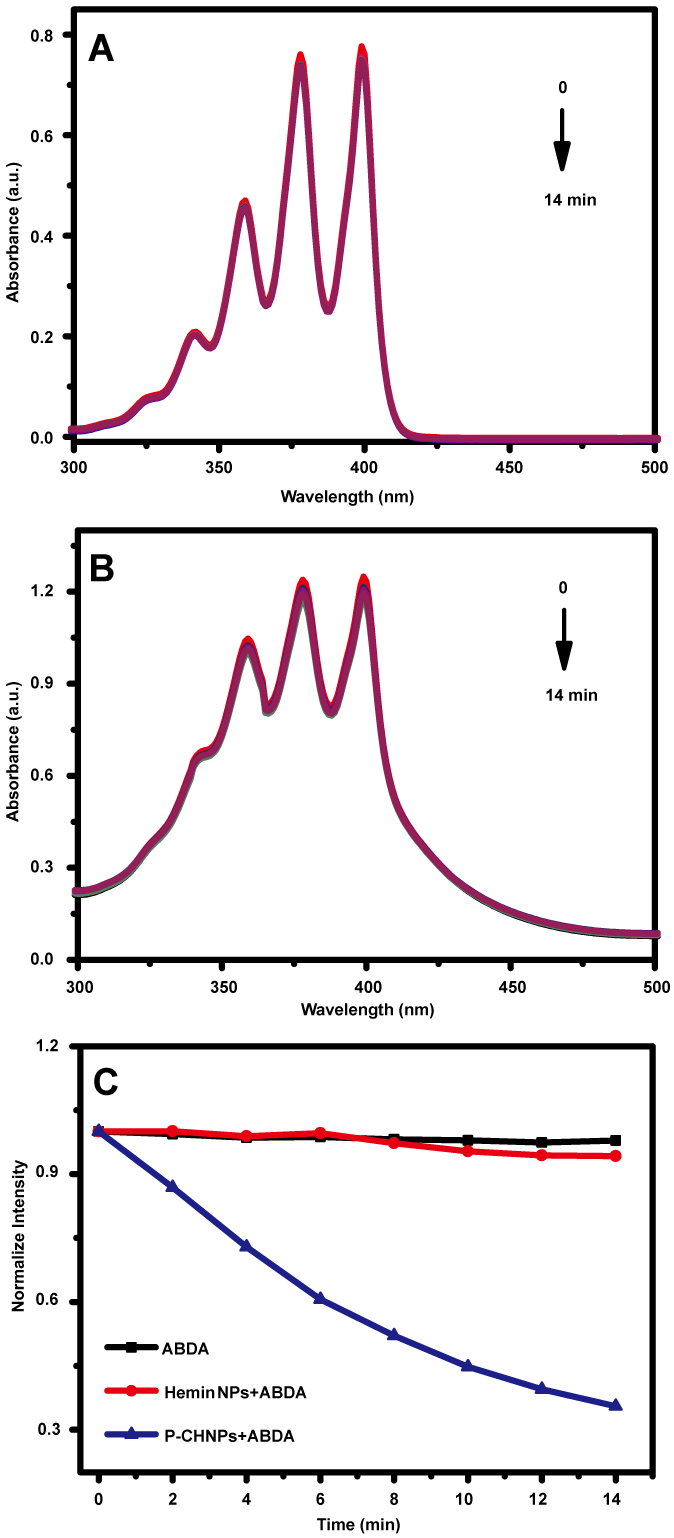


**Figure S7**. Time-sequenced absorption spectra of (A) ABDA solution and (B) mixture solution of ABDA and Hemin NPs under light irradiation. (C) Absorption variation of ABDA at 399 nm of the mixture solutions under light irradiation for different time. Light irradiation: 400-700 nm, 70 mW cm^-2^. [ABDA] = 50 μM, [P-CHNPs] = 50 μg mL^-1^. Hemin NPs: hemin was encapsulated with DSPE-mPEG.


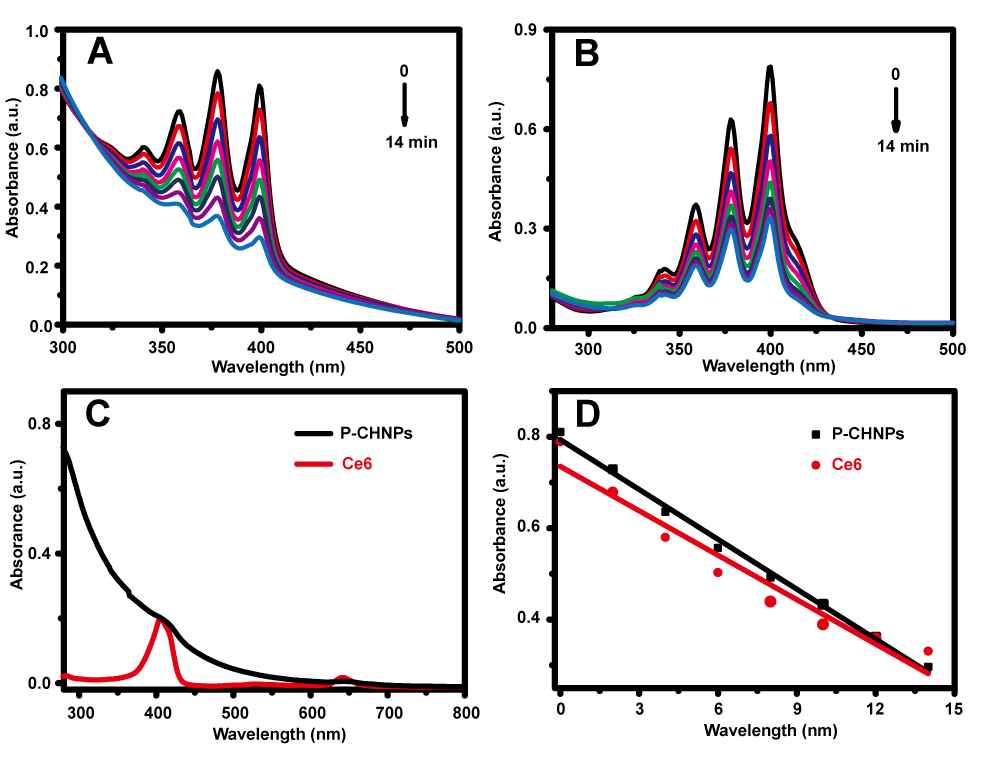


**Figure S8.** UV-vis spectra of ABDA mixed with (A) P-CHNPs and (B) Ce6 under light irradiation for different time. (C) The UV-vis absorption spectra of P-CHNPs and Ce6 solution. (D) The consumption rate of ABDA after light irradiation for different time. Light irradiation: 400 - 700 nm, 70 mW cm^-2^. [ABDA] = 50 µM, [Ce6] = 3.5 µM.


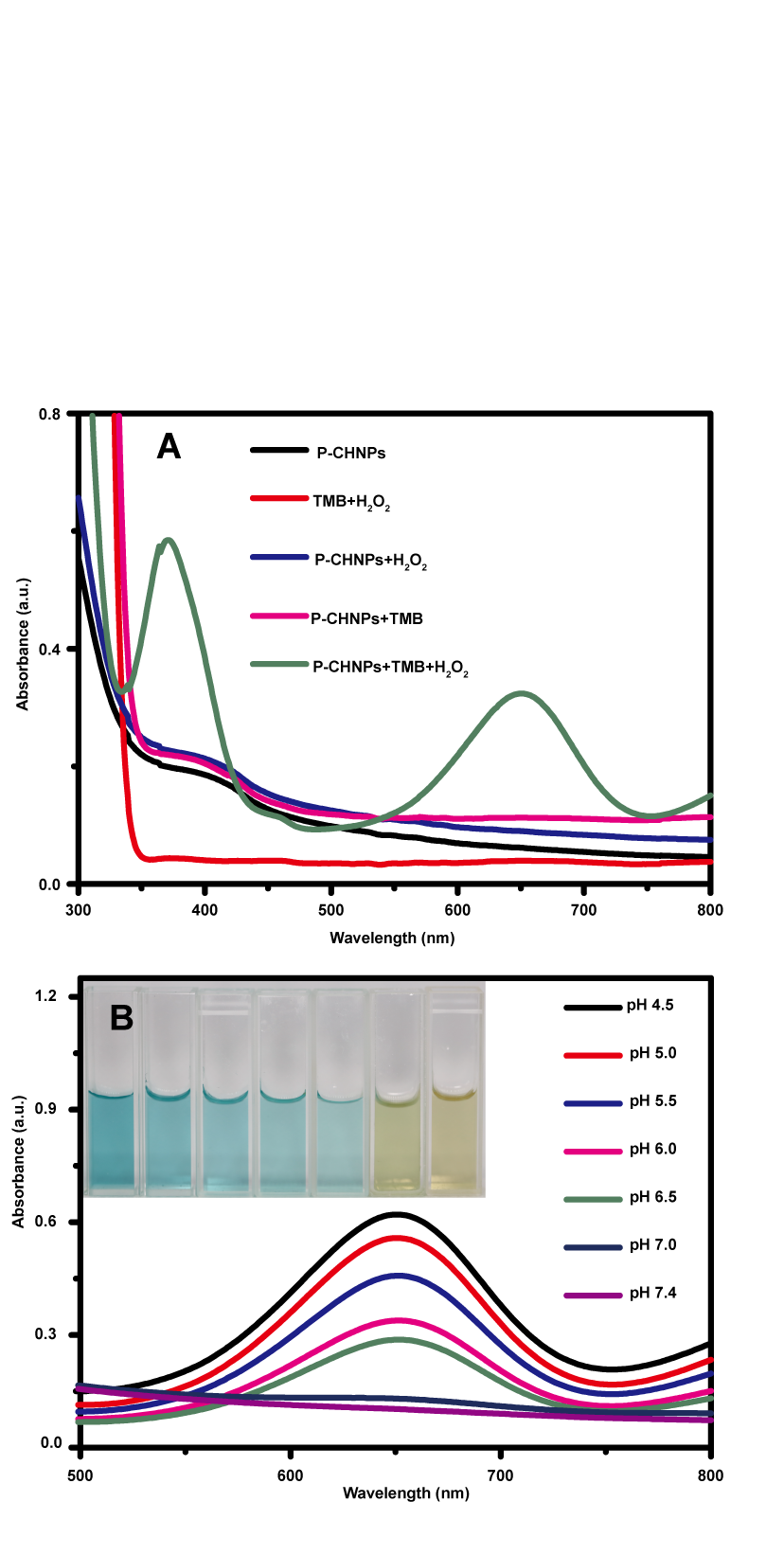


**Figure S9.** UV-vis absorption spectra of (A) the catalytic performance of P-CHNPs in different mixture solutions under pH 6.5 and (B) TMB, H_2_O_2_ and P-CHNPs mixture solutions with different pH values. Inset: Color changes of the solution as pH increment from 4.5 to 7. [TMB] = 800 μM, [P-CHNPs] = 50 μg mL^-1^, [H_2_O_2_] = 300 μM.


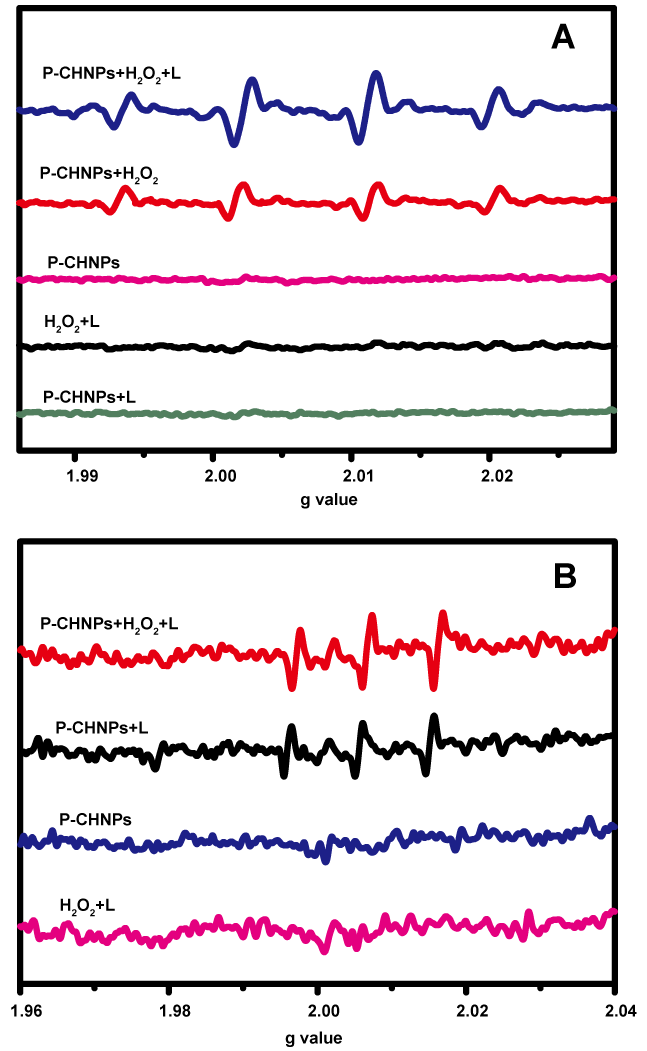


**Figure S10.** ESR spectra indicating the generation of (A) •OH and (B) ^1^O_2_ in the presence of P-CHNPs. The 5,5-dimethyl-1-pyrroline-Noxide (DMPO) were used as •OH trapping agent and 2,2,6,6-tetramethylpiperidine (TEMP) as ^1^O_2_ trapping agent. DMPO (600 μL reaction volume contained 7.5 μL DMPO solution), [TEMP] = 300 mM, [P-CHNPs] = 25 μg mL^-1^, [H_2_O_2_] = 600 μM; L: light irradiation (400 - 700 nm, 70 mW cm^-2^, 20 min).

**
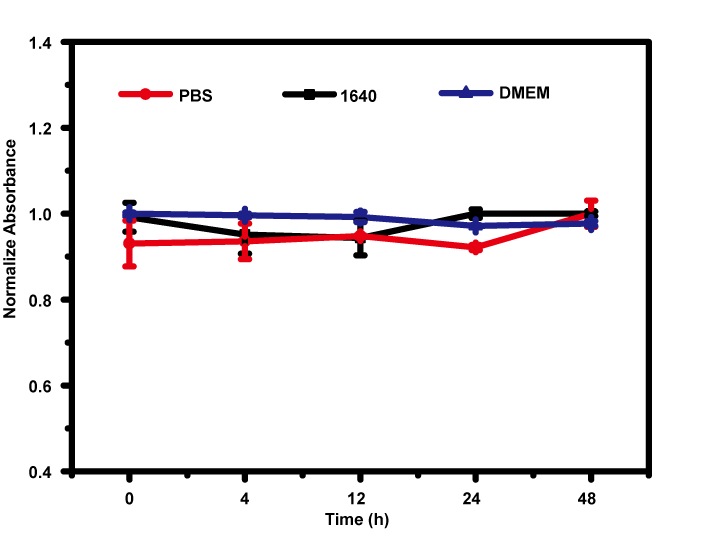
**

**Figure S11.** The stabilities of P-CHNPs incubated in PBS and cell medium (1640, DMEM) with serum for up to 48 h. [P-CHNPs] = 25 μg mL^-1^.


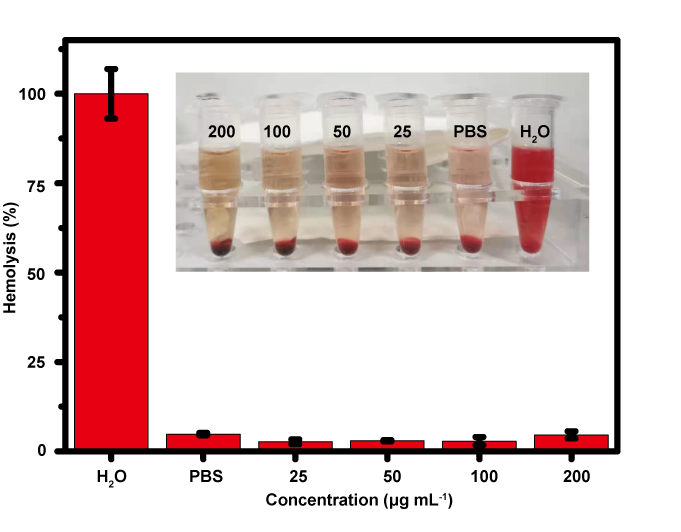


**Figure S12.** Hemolysis percentage of RBCs (4%, v/v) at various concentrations of P-CHNPs, inset: photos of RBCs cultured with P-CHNPs, PBS, H_2_O for 12 h.

**
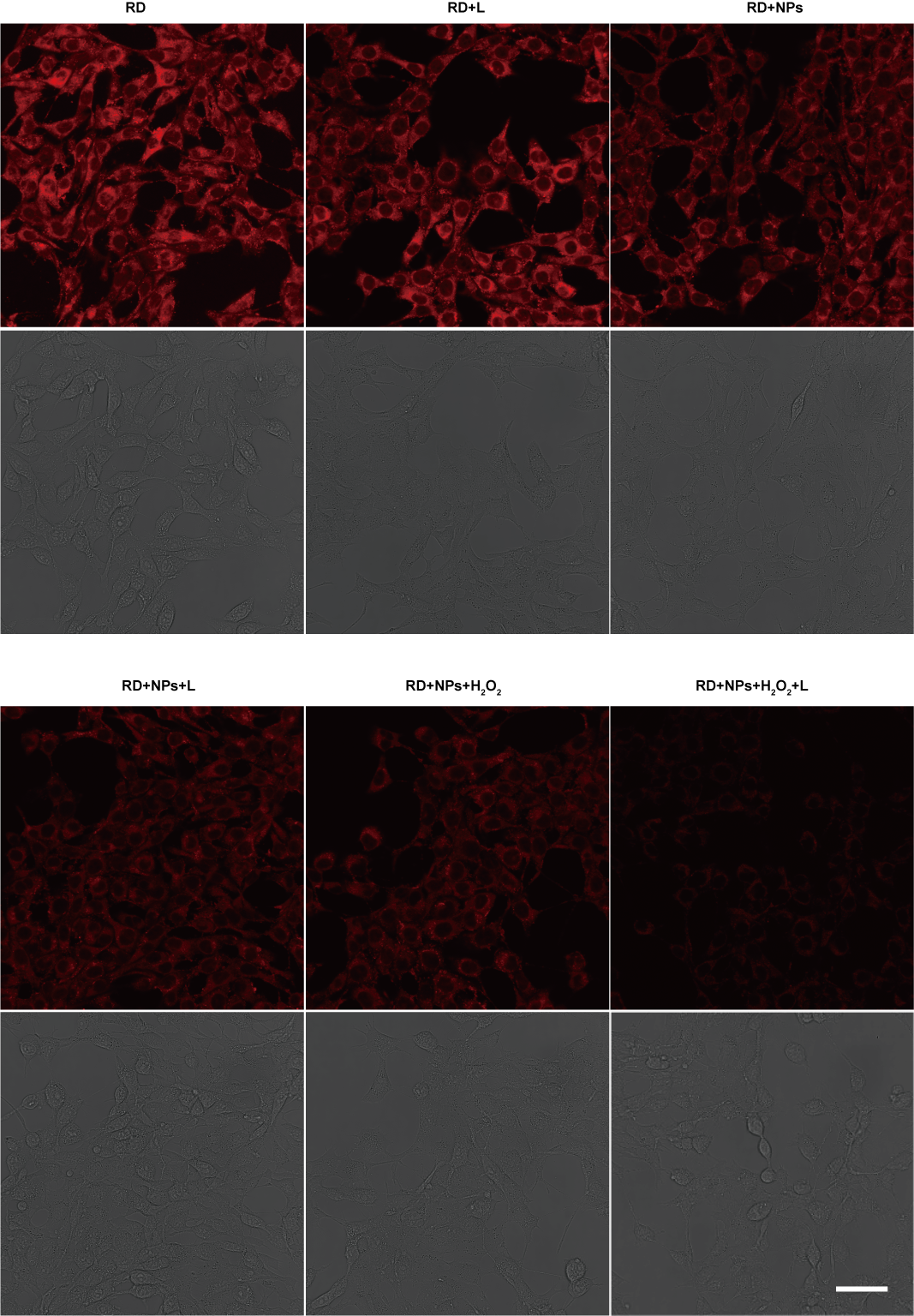
**

**Figure S13.** Fluorescence images of 4T1 cells with different treatments. [RDPP] = 10 μg mL^-1^, [P-CHNPs] = 200 μg mL^-1^, [H_2_O_2_] = 300 μM; L: light irradiation (400 - 700 nm, 70 mW cm^-2^, 20 min). NPs: P-CHNPs, RD: RDPP (Ex: 488 nm; Em: 560 - 700 nm), Scale bar: 200 μm.

**
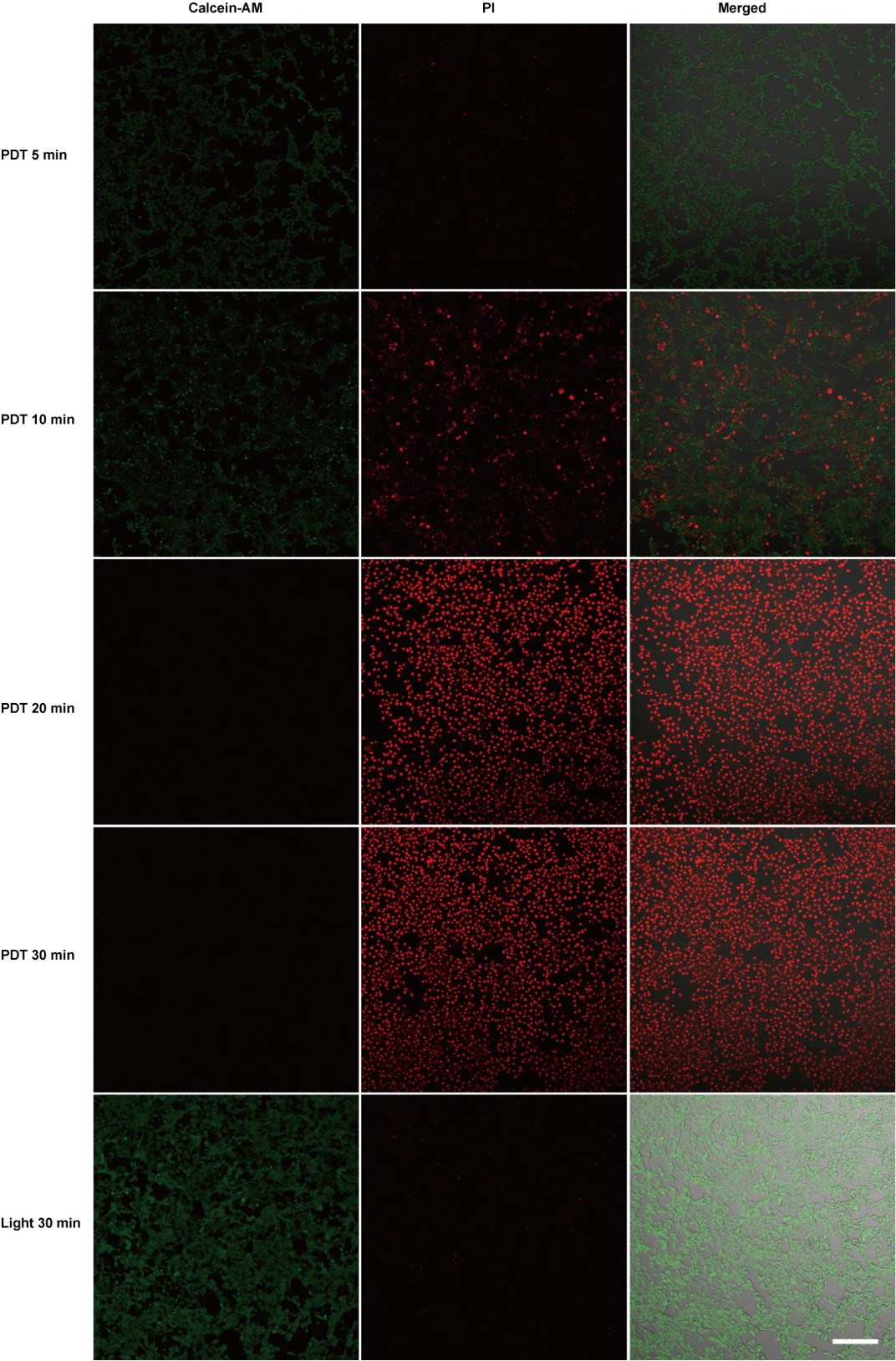
**

**Figure S14.** Fluorescence images of 4T1 cells stained by calcein-AM and PI after treated with/out P-CHNPs under different light irradiation conditions (5, 10, 20, 30 min). Light irradiation: 400 - 700 nm, 100 mW cm^-2^. [P-CHNPs] = 200 μg mL^-1^. Calcein-AM (Ex: 488 nm; Em: 505 - 525 nm) and PI (Ex: 552 nm; Em: 605 - 625 nm), [Calcein-AM] = 2 μM, [PI] = 2 μM. Scale bar: 200 μm.

**
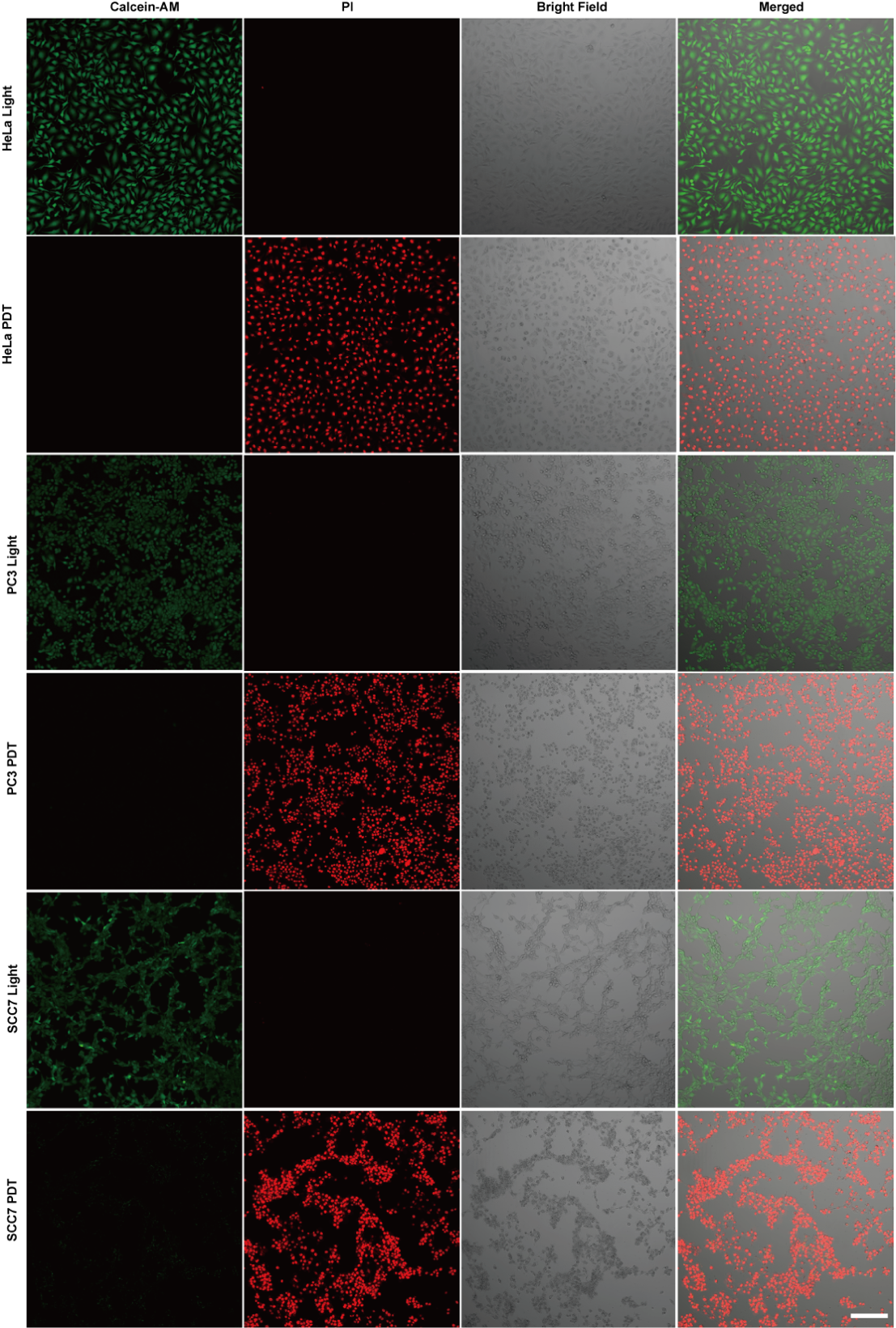
**

**Figure S15.** Cell viability of HeLa, PC3 and SCC7 cells treated with light irradiation (Light), and both light irradiation and P-CHNPs (PDT). Light irradiation: 400 - 700 nm, 100 mW cm^-2^, 20 min. [P-CHNPs] = 200 μg mL^-1^. Calcein-AM (Ex: 488 nm; Em: 505 - 525 nm) and PI (Ex: 552 nm; Em: 605 - 625 nm), [Calcein-AM] = 2 μM, [PI] = 2 μM. Scale bar: 200 μm.

**
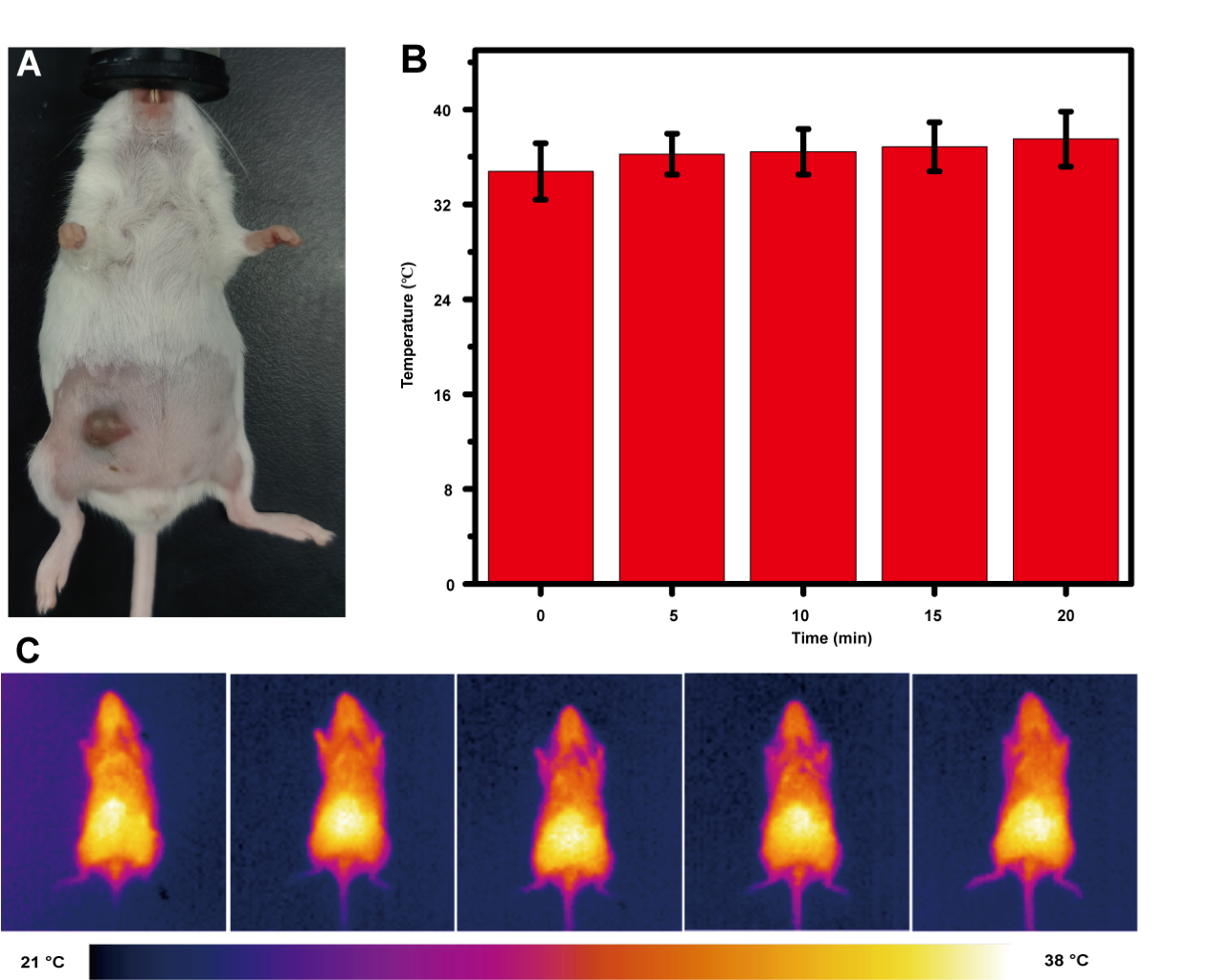
**

**Figure S16.** (A) Photographs of mice after intratumoral injection of P-CHNPs (8 mg kg^-1^). (B) Quantitative temperature of tumor tissues treated with P-CHNPs under light irradiation (n=4). (C) Time-sequenced IR thermal images of tumor-bearing mice treated with P-CHNPs under light irradiation. Light irradiation: 400 - 700 nm, 100 mW cm^-2^.


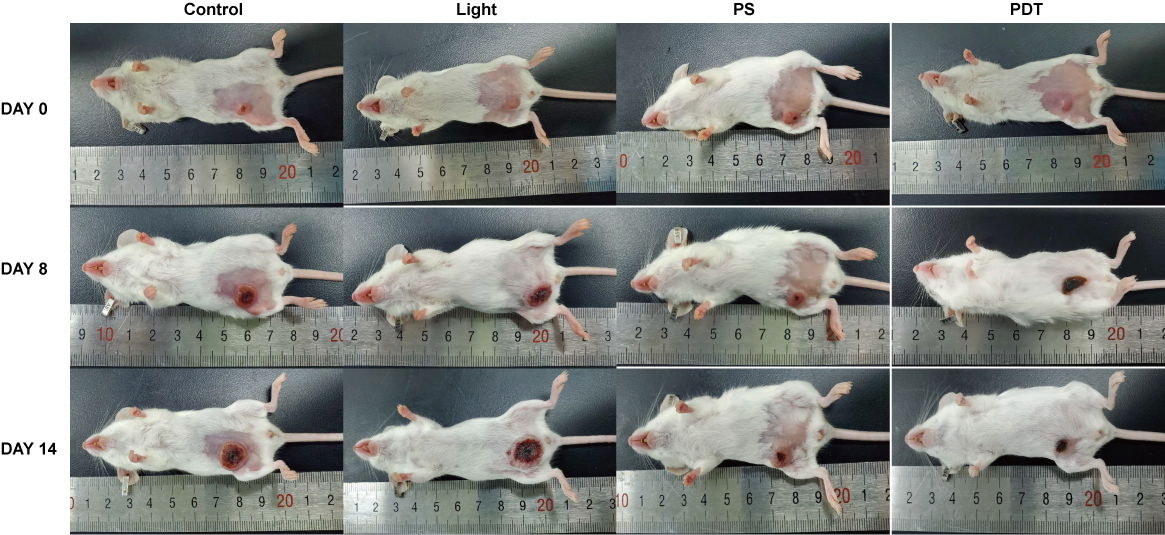


**Figure S17.** Photographs of mice after different treatments.


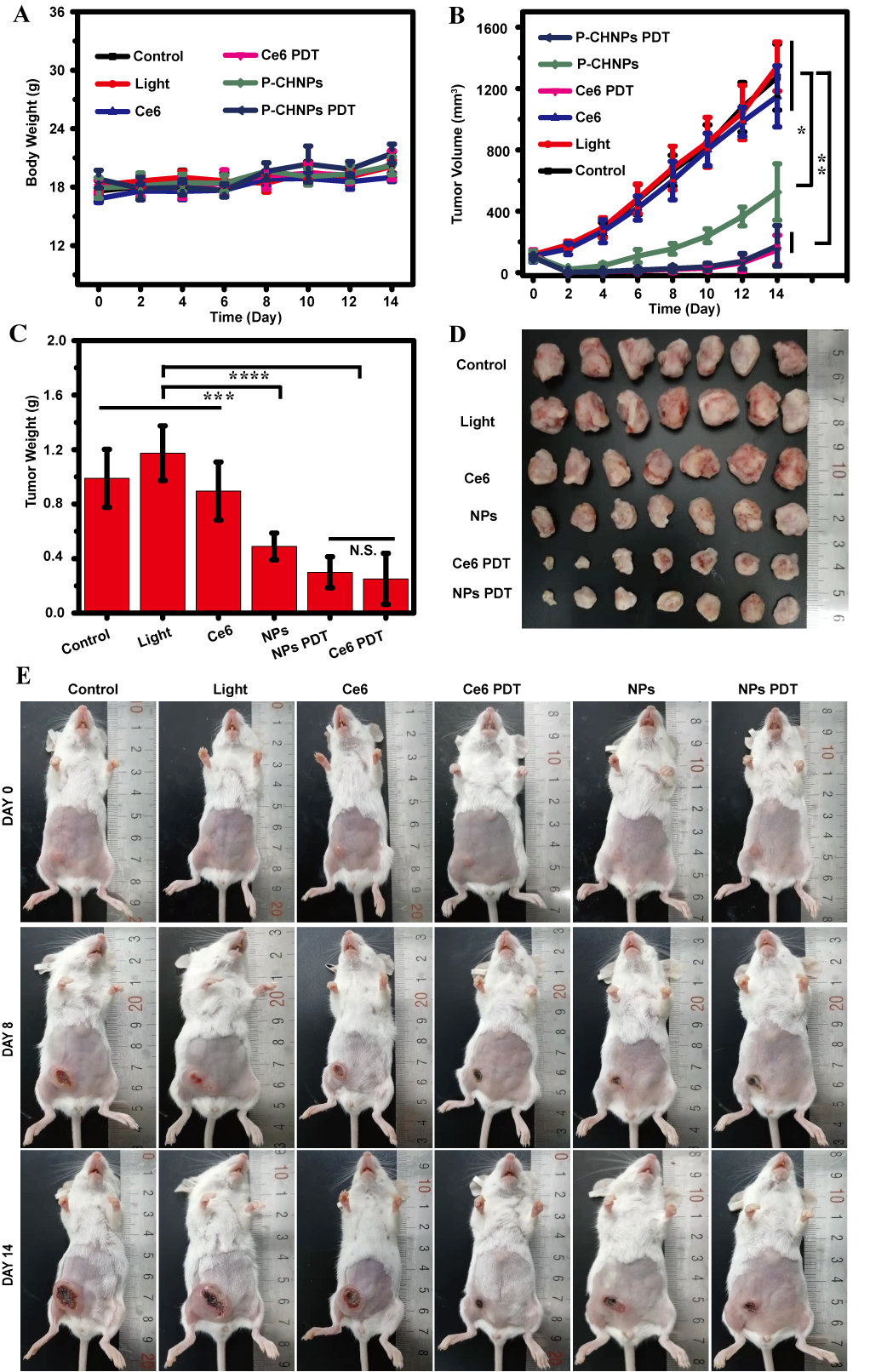


**Figure S18**. Comparation between the P-CHNPs based and Ce6 based in vivo photodynamic anticancer therapy effect. (A) Body weights of 4T1-tumor-bearing mice in all groups (n = 7). (B) Tumor growth curves of 4T1-tumor-bearing mice in different groups. Error bars were based on standard deviations (n = 7). (C) Statistical analysis of tumor weight on day 14 post-treatment (n = 7). (D) The image of excised tumors on day 14 post-treatment. (E) Photographs of mice after different treatments. [P-CHNPs] = 8 mg kg^-1^. [Ce6] = 5 mg kg^-1^. NPs: P-CHNPs, N.S.: no significant difference, * p < 0.05, ** P < 0.01, *** P < 0.001, **** P < 0.0001, n=7.

**
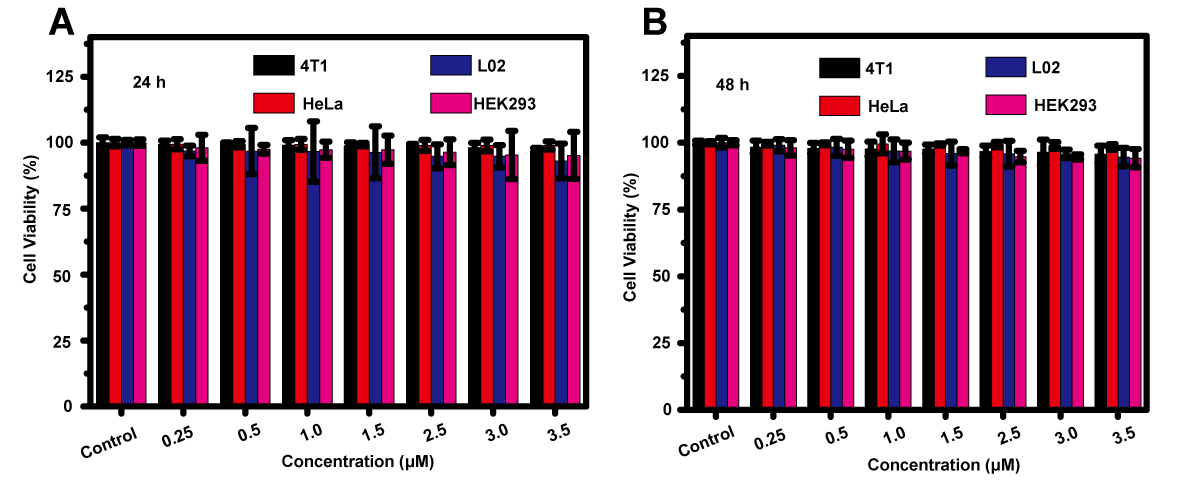
**

**Figure S19.** Cell viability of 4T1 and HeLa cancer cells, HEK293 and L02 normal cells treated with various concentrations of Ce6 for 24 and 48 h. Data were expressed as means ± s.d. (n = 3).


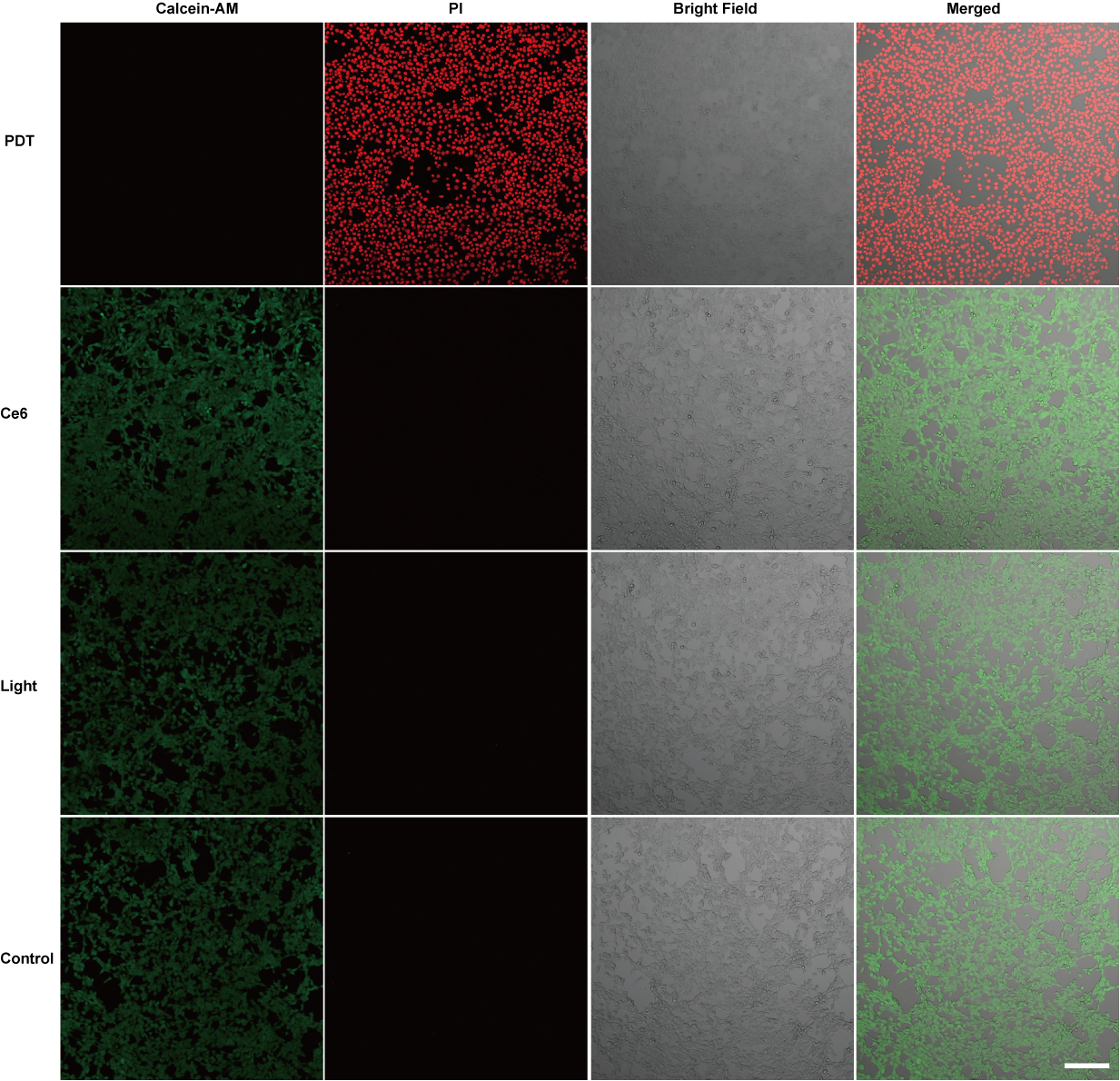


**Figure S20.** Fluorescence images of 4T1 cells stained by calcein-AM and PI after treated with nothing (Control), light irradiation (Light), Ce6 (PS) and both light irradiation and Ce6 (PDT). Light irradiation: 400 - 700 nm, 100 mW cm^-2^, 20 min. [Ce6] = 3.0 μM. Calcein-AM (Ex: 488 nm; Em: 505 - 525 nm) and PI (Ex: 552 nm; Em: 605-625 nm), [Calcein-AM] = 2 μM, [PI] = 2 μM. Scale bar: 200 μm.


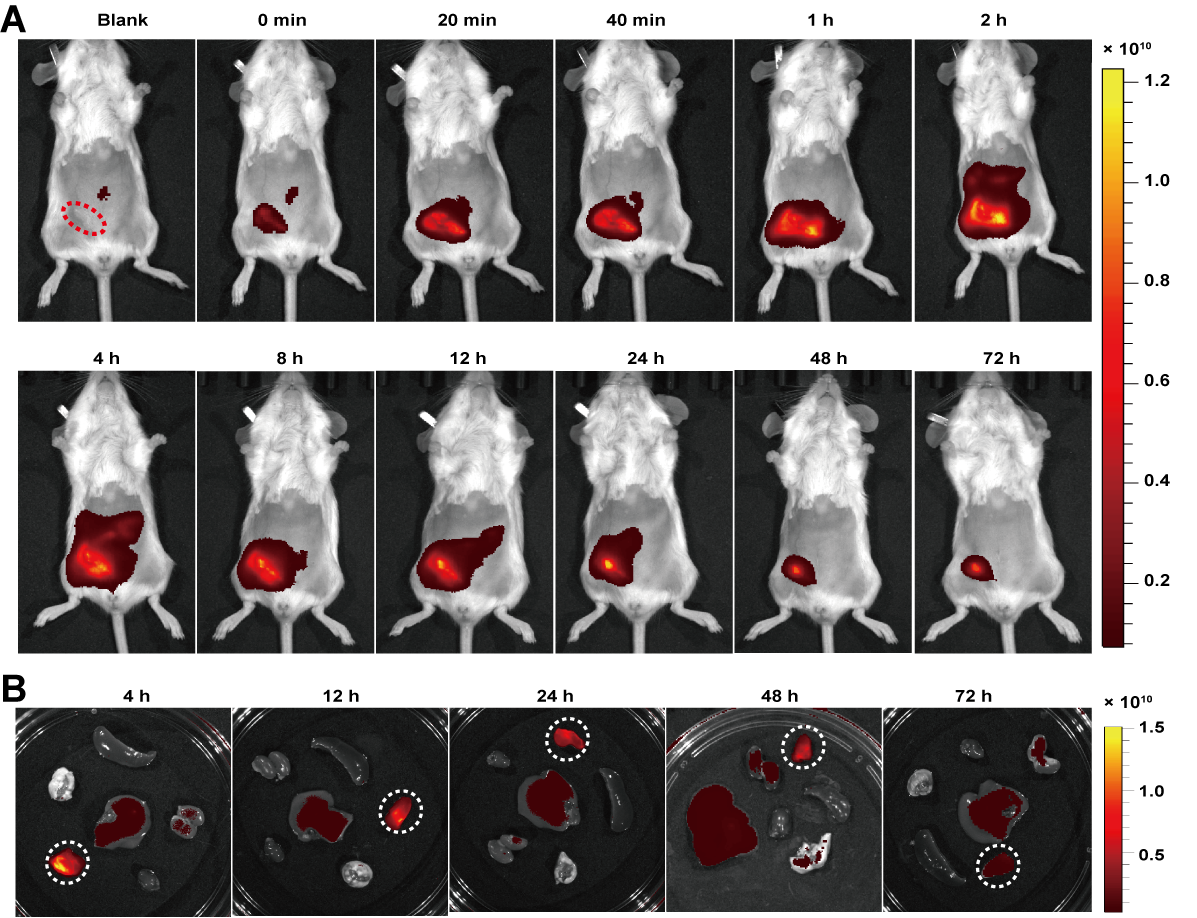


**Figure S21.** Time-sequenced (A) in vivo fluorescence images of Ce6 treated mice (5 mg kg^-1^) and (B) ex vivo fluorescence images of tumors and major organs excised from Ce6 treated mice (5 mg kg^-1^). The circle indicated the tumor.


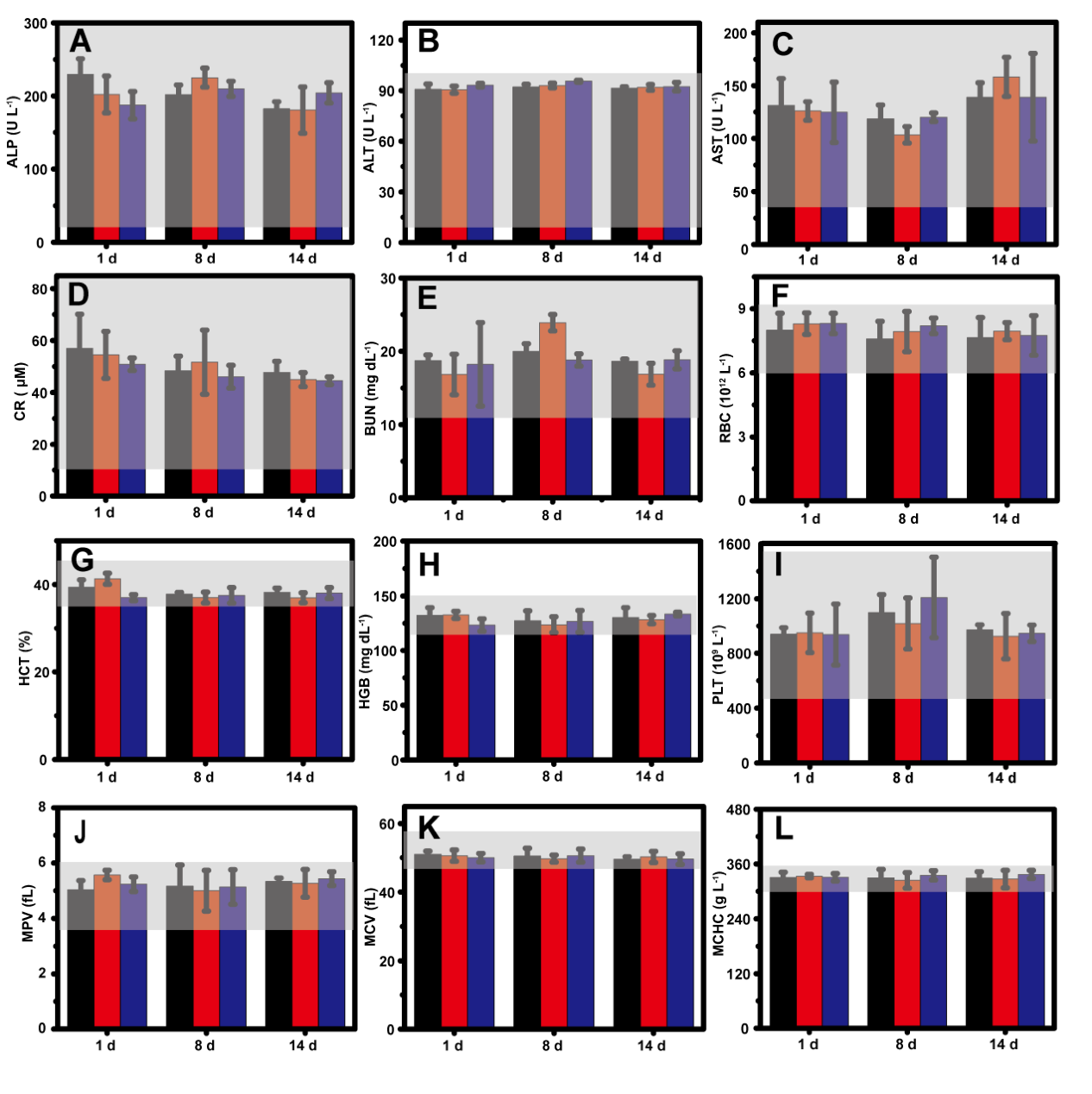


**Figure S22.** (A-E) Blood biochemistry tests and (F-L) hematology parameters of untreated mice (black), and mice treated with P-CHNPs alone (red), and mice treated with both P-CHNPs and light irradiation (blue) at day 1, 8, 14. [P-CHNPs] = 8 mg kg^-1^, light irradiation: 400 - 700 nm, 100 mW cm^-2^, 20 min. The reference range was marked in grey. Data were expressed as means ± s.d. (n = 3).


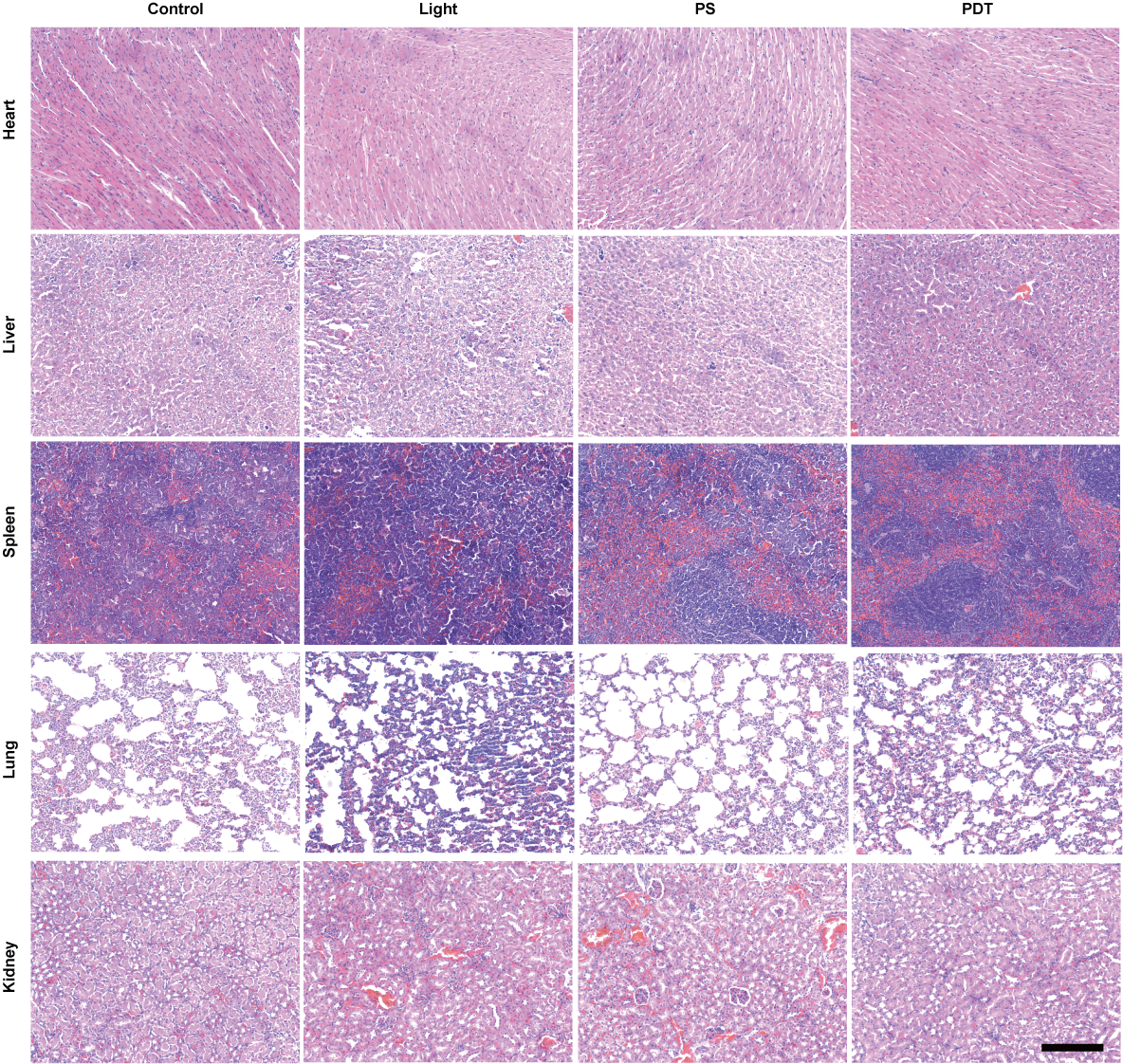


**Figure S23.** Histologic studies (H&E) on major organs of mice from different groups. Scale bar: 200 μm.

**Table S1.** Comparison of photodynamic anticancer therapy efficiency between the proposed P-CHNPs and previously reported PSs.

| **Strategy** | **PSs** | **ROS** | **Efficacy**  **(Inhibition rate)** | **Dose** | **Light conditions (Wavelength, power density, time)** | **Ref** |
| --- | --- | --- | --- | --- | --- | --- |
| PDT | Mesoporous Si-coated UCNs coloaded with ZnPc and MC540 | ^1^O_2_ | B16-F0 murine melanoma cells, 55% | 1.5 mg mL^-1^ | 980 nm, 2.5 W cm^-2^, 40 min | 3 |
|  |  |  | Subcutaneous melanoma tumors, 92% | 30 mg kg^-1^ | 980 nm, 415 mW cm^-2^, 120 min |  |
| PDT | UCNPs@TiO_2_ | ^1^O_2_ | HeLa cells, 75% | 800 μg mL^-1^ | 980 nm, 4.7 W cm^-2^, 30 min (5 min OFF after 10 min ON) | 4 |
|  |  |  | HeLa tumor,  77.8% | 50 mg kg^-1^ | 980 nm, 2.9 W cm^-2^, 30 min (5 min OFF after 10 min ON) |  |
| PDT | Upconversion nanoprobes anchored MnO_2_ nanosheet | ^1^O_2_ | 4T1 cells, 40% | 30 μg mL^-1^ | 980 nm, 1.5 W cm^-2^, 5 min (0.5 min OFF after 1 min ON); X-ray, 5 Gy, 5 min | 5 |
|  |  |  | 4T1 tumor,  93.3% | 80 mg kg^-1^ | 980 nm, 2W cm^-2^, 10 min (0.5 min OFF 1 min ON); X-ray, 8 Gy, 5 min |  |
| PDT | TiO_2_-UCNs | ^1^O_2_ | OSCC cells, 78% | 1mM | 980 nm, 2.1 W cm^-2^, 5.3 min | 6 |
|  |  |  | OSCC tumor, 85.7% | 30.3 mg kg^-1^ | 980 nm, 0.5 W cm^-2^, 33.3 min |  |
| PDT/Chemotherapy | Biomimetic MOF nanoplatform | ^1^O_2_ | 4T1 cells, 62.9% | 20 µg mL^-1^ (based on MOFs) | 660 nm, 0.1 W cm^-2^, 15 min | 7 |
|  |  |  | 4T1 tumor,  82% | 30 mg kg^-1^ (based on MOFs) | 660 nm, 0.1 W cm^-2^, 30 min |  |
| PDT/CDT | P-CHNPs | ^1^O_2_/•OH | 4T1 cells, 100% | 200 μg mL^-1^ | 400-700 nm  0.1 W cm^-2^, 20 min | This work |
|  |  |  | 4T1 tumor,  97.7% | 4 mg kg^-1^ | 400-700 nm  0.1 W cm^-2^, 20 min |  |

**Table S2.** Hematological parameters of the mice untreated, treated with P-CHNPs alone, treated with both P-CHNPs and light irradiation at day 1, 8, 14, and the reference ranges of normal mice.

| **Parameters** | **Day** | **Control** | **P-CHNPs** | **PDT** | **Reference** |
| --- | --- | --- | --- | --- | --- |
| ALP (U L^-1^) | 1 | 229.87 ± 21.09 | 202.14 ±25.31 | 187.31± 18.76 | 22.52-474.35 |
|  | 8 | 201.85 ± 13.05 | 225.17 ±13.06 | 209.72 ± 10.44 |  |
|  | 14 | 182.34 ± 9.82 | 180.61 ±31.70 | 204.15 ± 13.87 |  |
| ALT (U L^-1^) | 1 | 90.80 ± 3.05 | 90.55 ± 2.09 | 93.24 ± 1.07 | 10.06-96.47 |
|  | 8 | 92.11 ± 1.62 | 92.99 ± 1.39 | 95.62 ± 0.62 |  |
|  | 14 | 91.40 ± 0.89 | 91.90 ± 1.69 | 92.32 ± 2.48 |  |
| AST (U L^-1^) | 1 | 131.17 ± 25.53 | 126.07 ± 8.79 | 124.80 ± 28.49 | 36.31-235.48 |
|  | 8 | 118.60 ± 12.99 | 103.47 ± 7.78 | 120.07 ± 3.98 |  |
|  | 14 | 139.19 ± 13.54 | 158.39 ±18.56 | 139.11 ± 41.56 |  |
| CR  (μM) | 1 | 57.01 ± 13.12 | 54.48 ± 9.02 | 50.87 ± 2.43 | 10.91-85.09 |
|  | 8 | 48.43 ± 5.56 | 51.69 ± 12.34 | 46.07 ± 4.41 |  |
|  | 14 | 47.74 ± 4.26 | 44.96 ± 2.71 | 44.56 ± 1.49 |  |
| BUN  (mg dL^-1^) | 1 | 18.72 ± 0.78 | 16.84 ± 2.76 | 18.22 ± 5.68 | 10.81-34.74 |
|  | 8 | 19.98 ± 1.04 | 23.89 ± 1.11 | 18.81 ± 0.83 |  |
|  | 14 | 18.64 ± 0.30 | 16.87 ± 1.49 | 18.84 ± 1.23 |  |
| RBC  (10^12^ L^-1^) | 1 | 7.99 ± 0.79 | 8.30 ± 0.50 | 8.31 ± 0.48 | 6.36-9.42 |
|  | 8 | 7.59 ± 0.82 | 7.93 ± 0.94 | 8.20 ± 0.36 |  |
|  | 14 | 7.65 ± 0.93 | 7.95 ± 0.40 | 7.75 ± 0.92 |  |
| HCT (%) | 1 | 39.40 ± 1.69 | 41.33 ± 1.27 | 37.03 ± 0.68 | 34.6-44.6 |
|  | 8 | 37.17 ± 0.63 | 37.03 ± 1.23 | 37.53 ± 1.76 |  |
|  | 14 | 38.23 ± 0.97 | 37.0 ± 1.13 | 36.40 ± 3.61 |  |
| HGB  (g L^-1^) | 1 | 132.33 ± 6.94 | 132.67 ± 3.30 | 123.33 ± 5.73 | 110-143 |
|  | 8 | 127.33 ± 8.99 | 123.67 ± 7.32 | 126.67 ± 10.08 |  |
|  | 14 | 130.33 ± 8.99 | 128.33 ± 3.77 | 133.33 ± 1.70 |  |
| PLT  (10^9^ L^-1^) | 1 | 940.0 ± 47.34 | 949.67 ±144.99 | 937.67 ± 223.71 | 450-1590 |
|  | 8 | 1098.0 ±131.65 | 1019.0 ±187.21 | 1208.67 ±294.11 |  |
|  | 14 | 971.67 ± 37.19 | 925.0 ± 166.54 | 946.67 ± 60.79 |  |
| MPV (fL) | 1 | 5.03 ± 0.33 | 5.57 ± 0.17 | 5.23 ± 0.26 | 3.8-6.0 |
|  | 8 | 5.17 ± 0.76 | 5.0 ± 0.73 | 5.13 ± 0.62 |  |
|  | 14 | 5.33 ± 0.12 | 5.27 ± 0.50 | 5.43 ± 0.25 |  |
| MCV (fL) | 1 | 51.03 ± 0.90 | 50.60 ± 1.67 | 50.0 ± 1.23 | 48.2-58.3 |
|  | 8 | 50.50 ± 2.27 | 49.73 ± 1.03 | 50.60 ± 1.91 |  |
|  | 14 | 49.57 ± 0.74 | 50.23 ± 1.65 | 49.60 ± 1.55 |  |
| MCHC  (g L^-1^) | 1 | 330.33 ± 11.44 | 333.33 ± 3.86 | 330.67 ± 8.22 | 302-353 |
|  | 8 | 329.33 ± 18.66 | 324.0 ± 16.99 | 335.0 ± 10.03 |  |
|  | 14 | 329.00 ± 13.93 | 326.67 ±19.19 | 337.0 ± 8.98 |  |

**References**

1. Fery-Forgues, S. Lavabre, D. Are fluorescence quantum yields so tricky to measure? A demonstration using familiar stationery products. *J. Chem. Educ.* **76**, 1260-1264 (2014).

2. Wilkinson, F., Helman, W. P. Ross, A. B. Quantum yields for the photosensitized formation of the lowest electronically excited singlet state of molecular oxygen in solution. *J. Phys. Chem. Ref. Data* **22**, 113-262 (1993).

3. Idris, N. M. et al. In vivo photodynamic therapy using upconversion nanoparticles as remote-controlled nanotransducers. Nat. Med. **18**, 1580-1585 (2012).

4. Hou, Z. et al. UV-emitting upconversion-based TiO_2_ photosensitizing nanoplatform: near-infrared light mediated in vivo photodynamic therapy via mitochondria-involved apoptosis pathway. ACS Nano **9**, 2584-2599 (2015).

5. Fan, W. et al. Intelligent MnO_2_ nanosheets anchored with upconversion nanoprobes for concurrent pH‐/H_2_O_2_‐responsive UCL imaging and oxygen‐elevated synergetic therapy. Adv. Mater. **27**, 4155-4161 (2015).

6. Lucky, S. S. et al. Titania coated upconversion nanoparticles for near-infrared light triggered photodynamic therapy. ACS Nano **9**, 191-205 (2015).

7. Min, H. et al. Biomimetic metal-organic framework nanoparticles for cooperative combination of antiangiogenesis and photodynamic therapy for enhanced efficacy. Adv. Mater. **31**, 1808200 (2019).
